# Tailored Exercise Strategies and Mortality Among Breast Cancer Survivors

**DOI:** 10.1001/jamanetworkopen.2026.5177

**Published:** 2026-04-13

**Authors:** Jinani Jayasekera, Isaac J. Ergas, Jacob Schneider, Emma Tian, Kaitlyn M. Wojcik, Janise M. Roh, Jacqueline B. Vo, Lawrence H. Kushi, Oliver W.A. Wilson, Emma E. McGee

**Affiliations:** 1 Division of Intramural Research, National Institute on Minority Health and Health, Disparities, National Institutes of Health, Bethesda, Maryland; 2 Division of Research, Kaiser Permanente Northern California, Pleasanton; 3 Division of Cancer Epidemiology and Genetics, National Cancer Institute, Bethesda, Maryland; 4 Department of Epidemiology, Harvard T.H. Chan School of Public Health, Boston, Massachusetts; 5 Eric and Wendy Schmidt Center, Broad Institute, Massachusetts Institute of Technology and Harvard, Cambridge

## Abstract

**Importance:**

Guidelines recommend tailored (ie, individualized) exercise strategies for cancer survivors. However, there are limited data on the effects of these strategies on long-term mortality outcomes among breast cancer survivors.

**Objective:**

To estimate the effect of tailored exercise strategies on mortality for breast cancer survivors.

**Design, Setting, and Participants:**

In this cohort study, a target trial protocol was first specified to approximately mirror the Colon Health and Lifelong Exercise Change (CHALLENGE) trial among breast cancer survivors. Observational data from the Pathways Study were used to emulate this first target trial. These results were then extended by emulating a second target trial of more pragmatic, tailored exercise strategies applied to a broader population of breast cancer survivors. Women enrolled in the Kaiser Permanente Northern California health plan were recruited from January 2006 to December 2013, and were followed up through December 2021. Analyses were performed between September 2024 and September 2025.

**Exposures:**

Tailored exercise strategies adaptively modified based on evolving characteristics.

**Main Outcomes and Measures:**

All-cause and breast cancer–specific mortality.

**Results:**

In the first target trial, there were 959 eligible women (mean [SD] age, 58.3 [12.5] years) and 183 deaths. Compared with a strategy similar to the health education intervention of the CHALLENGE trial, an aerobic exercise strategy was associated with an 8.0 (95% CI, 3.4-13.3)–percentage point lower 8-year all-cause mortality risk, which was compatible with the CHALLENGE trial (7.1 [95% CI, 1.8-12.3]–percentage point lower risk). In the second target trial, there were 2107 eligible women (mean [SD] age, 60.1 [12.1] years) and 321 deaths. Estimated 10-year all-cause mortality ranged from 18.1% to 21.2%. Breast cancer–specific mortality ranged from 7.6% to 10.0%. Compared with no intervention, engaging in a tailored strategy requiring an increase of 60 minutes of vigorous or 120 minutes of moderate aerobic exercise per week was associated with a 3.1 (95% CI, 2.0-4.6)–percentage point lower 10-year all-cause mortality risk and a 2.4 (95% CI, 1.2-3.5)–percentage point lower 10-year breast cancer–specific mortality risk.

**Conclusions and Relevance:**

In this cohort study using a target trial emulation design, tailored exercise strategies were associated with reductions in 10-year mortality among breast cancer survivors. A randomized trial is warranted to confirm these findings.

## Key Points

**Question:**

How do tailored (ie, individualized) exercise strategies affect mortality among breast cancer survivors?

**Findings:**

This cohort study with a target trial emulation design initially included 959 women and approximately replicated the results of the Colon Health and Lifelong Exercise Change randomized trial among breast cancer survivors. These findings were then extended to show that more pragmatic, tailored strategies requiring adaptive increases in exercise levels were associated with lower 10-year all-cause and breast cancer–specific mortality among a broader group of 2107 breast cancer survivors.

**Meaning:**

These results suggest that clinicians may consider pragmatic, tailored strategies requiring increases in exercise levels that are adapted to the individual characteristics of breast cancer survivors.

## Teaser

This cohort study estimates the association of pragmatic, tailored exercise strategies with long-term mortality outcomes in breast cancer survivors.

## Introduction

Guidelines recommend that clinicians offer individualized (ie, tailored) exercise prescriptions to individuals diagnosed with cancer (cancer survivors).^1,2^ For instance, the American Cancer Society recommends exercise strategies that include “appropriate and tailored modifications” to accommodate diagnoses and treatment-related issues.^1^ Although the Colon Health and Lifelong Exercise Change (CHALLENGE) randomized trial estimated a strong protective effect of a structured recreational exercise intervention on 8-year mortality among 889 colon cancer survivors,^3^ to our knowledge, no randomized trial has evaluated the effects of tailored exercise strategies on long-term mortality outcomes among breast cancer survivors. Breast cancer survivors often encounter challenges due to fatigue, pain, lymphedema, and increased cardiovascular risk that may limit their ability to engage in high levels of exercise.^4,5,6^ As a result, it is important to quantify the effects of pragmatic, tailored exercise strategies that require gradual increases in exercise levels and that allow for modifications based on evolving clinical characteristics. Furthermore, to inform clinical practice, it is also important to estimate the effects of these strategies in broad populations of breast cancer survivors.

To address these gaps, we aimed to estimate the effects of pragmatic, tailored exercise strategies on long-term mortality outcomes for a broad population of breast cancer survivors. To accomplish this aim, we used observational data from the Pathways Study,^7^ a prospective cohort of women diagnosed with breast cancer, to build on the findings of the CHALLENGE trial.^3^ First, we specified the protocol of a hypothetical, pragmatic randomized trial that approximately mirrored the CHALLENGE trial but was conducted among breast cancer survivors. We refer to this hypothetical trial as a target trial.^8^ Second, we used observational data from the Pathways Study to emulate this first target trial. Third, we used data from the Pathways Study to extend the results of the CHALLENGE trial by emulating a second target trial that aimed to estimate the effects of more pragmatic, tailored exercise strategies in a broader population of breast cancer survivors.^8,9^ The overarching goal of this study was to provide novel data to support individualized exercise recommendations for breast cancer survivors in clinical settings.

## Methods

This cohort study was approved by the National Institutes of Health Institutional Review Board and was considered exempt research based on use of deidentified preexisting data. This study follows the Transparent Reporting of Studies Emulating a Target Trial (TARGET) reporting guidelines (eMethods 2 in Supplement 1).^32^

### Target Trial 1

#### Specification 

Our initial step was to specify the protocol of a target trial among breast cancer survivors that mirrored, to the extent possible, the CHALLENGE trial. eTable 1 in Supplement 1 outlines the protocol of this target trial.

#### Eligibility Criteria

Briefly, individuals are eligible for the target trial if they meet the following criteria: (1) women 21 years or older who are members of the Kaiser Permanente Northern California (KPNC) health care system; (2) diagnosed with incident stage II or III invasive breast cancer between 2005 and 2013; (3) no substantial comorbid condition that could preclude participation in an exercise program, defined as a weighted Elixhauser Comorbidity Index of 14 or greater during the 12 months before diagnosis^10^ or swelling that interfered with exercise within the past 6 months; (4) currently engaging in less than the equivalent of 150 minutes per week of moderate- to vigorous-intensity recreational aerobic exercise; and (5) no prior history of invasive cancer.

#### Exercise Strategies

Each eligible individual is randomly assigned to an exercise strategy that approximately mirrors the recreational aerobic exercise levels achieved under (1) the health education intervention and (2) the recreational aerobic exercise intervention of the CHALLENGE trial (eTable 1 in Supplement 1).^3^ Interventions are discontinued if and when an individual experiences a disease event, defined as disease recurrence or second primary breast cancer. Women must also respond to follow-up questionnaires administered approximately every 2 years to update information on exercise and other clinical factors.

#### Outcomes, Follow-Up, and Causal Contrasts

The outcome of interest is all-cause mortality. Follow-up begins at assignment to an exercise strategy (baseline) and continues until the outcome, loss to follow-up (defined as questionnaire nonresponse), 10 years, or administrative end of follow-up (December 2021), whichever occurs first. The causal contrasts of interest are the intention-to-treat effect and the per-protocol effect of adhering to the strategies throughout the follow-up.

### Emulation

Ideally, we would conduct the randomized target trial outlined in the previous section. However, in the absence of randomized trial data, we used observational data from the Pathways Study, a prospective cohort of women with breast cancer enrolled in the KPNC health plan, to emulate the design and analysis of this trial.

Briefly, women in the Pathways Study completed a baseline questionnaire within a median (range) of approximately 2 (0.7-17.8) months after breast cancer diagnosis.^7,11^ Women were recruited from January 2006 to March 2013, and have since been followed up via electronic health record linkage and periodic questionnaires. The Arizona Activity Frequency Questionnaire^12^ was used to measure self-reported exercise at baseline and during follow-up (eTable 2 in Supplement 1). Self-reported race and ethnicity were obtained on the baseline questionnaire.^7^ Deaths were ascertained from family members, medical records, and KPNC mortality files, which include data from KPNC, the state of California, the Social Security Administration, and the National Death Index.

We assumed assignment to an exercise strategy within the Pathways Study was as if randomized conditional on covariates available on the baseline questionnaire or KPNC clinical records. We estimated an observational analogue only of the per-protocol effect because an analogue of the intention-to-treat effect would not be very informative if nonadherence to the exercise strategies was high, which is common in behavioral interventions.^13^

Women in the Pathways Study provided informed consent to participate in data collection and longitudinal follow-up. The Pathways Study was approved by the KPNC Institutional Review Board.

### Target Trial 2

In the second target trial, we used the Pathways data to emulate a trial in which we considered several extensions that allowed us to assess the effects of more pragmatic exercise strategies in a broader population of breast cancer survivors (eTable 1 in Supplement 1). These extensions included (1) allowing eligible women to be diagnosed with stage I breast cancer; (2) allowing eligible women to have a prior history of cancer; (3) changing the strategies to consider tailored strategies that required women to increase total aerobic exercise beyond their normal levels for 8 years, unless they developed a serious comorbid condition (defined based on exercise guidelines for cancer survivors and the available observational data)^14,15^; (3) estimating risks over 10 years; and (5) additionally estimating effects on breast cancer–specific mortality. To evaluate which, if any, of these modifications most strongly affected the estimates, we applied each extension sequentially in addition to evaluating them simultaneously.

### Statistical Analysis

In both target trials, we conducted per-protocol analyses to estimate the risks that would have been observed had everyone followed each strategy throughout the entire follow-up with no loss to follow-up. We used the parametric g-formula,^16,17^ a generalization of standardization that has previously been used to estimate per-protocol effects of lifestyle strategies.^18,19,20,21,22,23,24,25,26,27,28,29,30^ Risks estimated via the parametric g-formula were used to construct adjusted risk curves and were compared under different exercise strategies via risk differences and ratios at 8 years (target trial 1) or 10 years (target trial 2). In the first target trial, we also estimated hazard ratios over 8 years for comparison with the CHALLENGE trial.^3^ Percentile-based 95% CIs were generated using a nonparametric bootstrap with 500 resamples. Details on the covariates and models are provided in eTable 3 in Supplement 1. Additional information is provided in eMethods 1 in Supplement 1.

#### Sensitivity Analyses

We conducted several sensitivity analyses to evaluate the robustness of our results to key assumptions (eMethods 1 and eTable 4 in Supplement 1). These analyses included changing the outcome to questionnaire nonresponse, a negative control outcome that we hypothesized would not be directly affected by exercise but could be similarly confounded.^31^

#### Other Analyses

We also estimated the effects of tailored strategies requiring increases in exercise levels among women with any baseline level of exercise. Finally, for comparison with prior observational studies, we estimated outcomes associated with aerobic exercise strategies that were not tailored to evolving characteristics and that required individuals to be insufficiently active, active, or highly active compared with minimally active (eMethods 1 in Supplement 1).

Analyses were conducted using SAS software, version 9.4 (SAS Institute) with the GFORMULA macro and R software, version 4.4.0 (R Foundation for Statistical Computing). All analyses were performed between September 2024 and September 2025.

## Results

### Target Trial 1

A total of 959 women (mean [SD] age, 58.3 [12.5] years; 25 [2.6%] American Indian or Alaskan Native, 123 [12.8%] Asian, 136 [14.2%] Hispanic, 106 [11.3%] non-Hispanic Black, 561 [58.5%] non-Hispanic White, and 8 [0.8%] Pacific Islander) were eligible for the first target trial that approximately mirrored the CHALLENGE trial (Table 1; eFigure 1 in Supplement 1). Most participants were postmenopausal (647 [67.5%]), college educated (745 [77.7%]) women with obesity (437 [45.6%]) and were diagnosed with hormone receptor–positive (759 [79.1%]), stage II (709 [73.9%]) breast cancer.

**Table 1.** Baseline Characteristics of Eligible Individuals for the 2 Target Trial Emulations Using Observational Data From the Pathways Study (2005-2021)

Characteristic	No. (%) of participants^a^	
	Target trial 1 (n = 959)	Target trial 2 (n = 2107)
Age, mean (SD), y	58.3 (12.5)	60.1 (12.1)
Postmenopausal	647 (67.5)	1550 (73.5)
Race and ethnicity		
American Indian or Alaskan Native	25 (2.6)	41 (1.9)
Asian	123 (12.8)	287 (13.6)
Hispanic	136 (14.2)	272 (12.9)
Non-Hispanic Black	106 (11.3)	192 (9.1)
Non-Hispanic White	561 (58.5)	1303 (61.8)
Pacific Islander	8 (0.8)	12 (0.6)
Educational attainment		
High school or less	214 (22.3)	445 (21.1)
Some college	358 (37.3)	792 (37.6)
College graduate	236 (24.6)	534 (25.3)
Postcollege	151 (15.7)	336 (15.9)
Annual income, $		
<25 000	100 (10.4)	247 (11.7)
25 000 to <50 000	205 (21.4)	466 (22.1)
50 000 to <90 000	283 (29.5)	593 (28.1)
≥90 000	241 (25.1)	528 (25.1)
Unknown	130 (13.6)	273 (13.0)
Aerobic exercise, median (IQR)		
Moderate aerobic exercise, min/wk	90.0 (26.3-202.5)	90 (30.0-202.5)
Vigorous aerobic exercise, min/wk	0.0 (0.0-33.8)	0.0 (0.0-33.8)
Total moderate or vigorous aerobic exercise, MET-min/wk	1772.6 (1101.8-2729.3)	1771.9 (1125.0-2730.0)
Recreational moderate or vigorous aerobic activity, MET-min/wk	135.0 (0.0-360.0)	135.0 (0.0-360.0)
≥1 Muscle strengthening activity per week	10 (1.0)	37 (1.8)
BMI		
Underweight (<18.5)	11 (1.1)	25 (1.2)
Normal weight (≥18.5 to <25)	224 (23.4)	519 (24.6)
Overweight (≥25 to <30)	287 (29.9)	617 (29.3)
Obese or morbidly obese (≥30)	437 (45.6)	946 (44.9)
Smoking status		
Current smoker	539 (56.2)	1153 (54.7)
Quit ≤1 y ago	349 (36.4)	811 (38.5)
Never smoker or quit >1 y ago	71 (7.4)	143 (6.8)
Weighted Elixhauser Comorbidity Index		
<0	281 (29.3)	603 (28.6)
0-1	510 (53.2)	1104 (52.4)
≥2	168 (17.5)	400 (19.0)
AJCC stage		
Stage I	NA	1117 (53.0)
Stage II	709 (73.9)	728 (34.6)
Stage III	250 (26.1)	262 (12.4)
Lymph node positive	648 (67.6)	677 (32.1)
Hormone receptor status		
ER and PR positive	570 (59.4)	1347 (63.9)
ER or PR positive	189 (19.7)	413 (19.6)
ER and PR negative	200 (20.9)	347 (16.5)
*ERBB2 *(previously* HER2/neu*) status		
Positive	160 (16.7)	274 (13.0)
Negative	770 (80.3)	1752 (83.2)
Unknown	29 (3.0)	81 (3.8)
Year of diagnosis (calendar year)		
2005-2007	367 (38.3)	811 (38.5)
2008-2010	400 (41.7)	857 (40.7)
2011-2013	192 (20.0)	439 (20.8)
Treated with hormonal therapy^b^	686 (71.5)	1527 (72.4)
Treated with chemotherapy^b^	704 (73.4)	972 (46.1)
Treated with radiation therapy^b^	263 (27.4)	896 (42.5)
Surgery type^b^		
No surgery	54 (5.6)	67 (3.2)
Lumpectomy	422 (44.0)	1246 (59.1)
Mastectomy	483 (50.4)	794 (37.7)

Abbreviations: AJCC, American Joint Committee on Cancer; BMI, body mass index (calculated as weight in kilograms divided by the square of height in meters); ER, estrogen receptor; MET, metabolic equivalent of task; NA, not applicable; PR, progesterone receptor.

^a^ Unless otherwise indicated.

^b^ Breast cancer treatment and surgery data include initial treatments received during the first 12 months after diagnosis.

#### All-Cause Mortality

**Figure 1.**
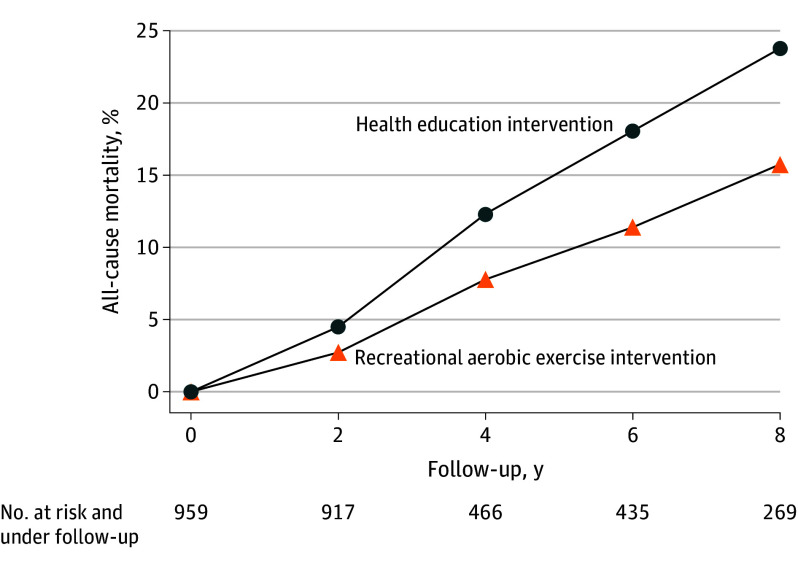
Estimated Risk Curves for All-Cause Mortality Under Different Exercise Strategies in the First Target Trial Emulation, Pathways Study (2005-2021) Under these strategies, eligible individuals were required to achieve exercise levels similar to those of the health education or recreational aerobic exercise interventions of the Colon Health and Lifelong Exercise Change (CHALLENGE) trial.^3^

During the 8-year follow-up, 183 deaths occurred (eTable 5 in Supplement 1). The observed risk of all-cause mortality was 24.2%. Using the parametric g-formula, the estimated 8-year risk of all-cause mortality under no intervention was 23.7% (95% CI, 20.1%-26.9%). The estimated 8-year all-cause mortality risk under a strategy that attempted to mirror the exercise levels achieved under the recreational aerobic exercise intervention of the CHALLENGE trial was 15.8% (95% CI, 9.6%-21.1%). Compared with a strategy that attempted to mirror exercise levels achieved under the health education intervention, this strategy was associated with an 8.0 (95% CI, 3.4-13.3)–percentage point lower 8-year risk of all-cause mortality (Figure 1 and Table 2). A similar contrast in the CHALLENGE trial was compatible with these estimates (7.1 [95% CI, 1.8-12.3]–percentage point lower risk).

**Table 2.** Estimated 8-Year Risks of All-Cause Mortality Under Different Exercise Strategies in the CHALLENGE Trial and in the First Target Trial Emulation Using Observational Data From the Pathways Study (2005-2021)^a^

Exercise strategy^b^	CHALLENGE trial (n = 889)			Target trial 1 (n = 959)		
	8-y Risk, % (95% CI)	Risk difference, % (95% CI)	HR (95% CI)	8-y Risk, % (95% CI)	Risk difference, % (95% CI)	HR (95% CI)
Health education intervention	16.8 (NR)	0 [Reference]	1 [Reference]	23.8 (20.0 to 26.9)	0 [Reference]	1 [Reference]
Recreational aerobic exercise intervention	9.7 (NR)	−7.1 (−12.3 to −1.8)	0.63 (0.43 to 0.94)	15.8 (9.6 to 21.1)	−8.0 (−13.3 to −3.4)	0.63 (0.39 to 0.85)

Abbreviations: CHALLENGE, Colon Health and Lifelong Exercise Change; HR, hazard ratio; NR, not reported.

^a^ Estimates were obtained using the parametric g-formula, which included baseline covariates (age, race and ethnicity, educational attainment, smoking status, menopausal status, weighted Elixhauser Comorbidity Index, cancer stage, nodal status, hormone receptor status, year of diagnosis, initial treatment with hormonal therapy, initial treatment with chemotherapy, initial treatment with radiotherapy, and initial surgery type) and time-varying covariates (body mass index, muscle-strengthening exercise, recreational aerobic exercise, and development of disease recurrence or second primary breast cancer).

^b^ Under these strategies, eligible individuals in the target trial were required to achieve exercise levels similar to those of the health education or recreational aerobic exercise interventions of the CHALLENGE trial.^3^

### Target Trial 2

A total of 2107 women (mean [SD] age, 60.1 [12.1] years; 41 [1.9%] American Indian or Alaskan Native, 287 [13.6%] Asian, 272 [12.9%] Hispanic, 192 [9.1%] non-Hispanic Black, 1303 [61.8%] non-Hispanic White, and 12 [0.6%] Pacific Islander) were eligible for the second target trial (eFigure 2 in Supplement 1). Of these women, 1117 (53.0%) were diagnosed with stage I breast cancer (Table 1).

#### All-Cause Mortality

**Figure 2.**
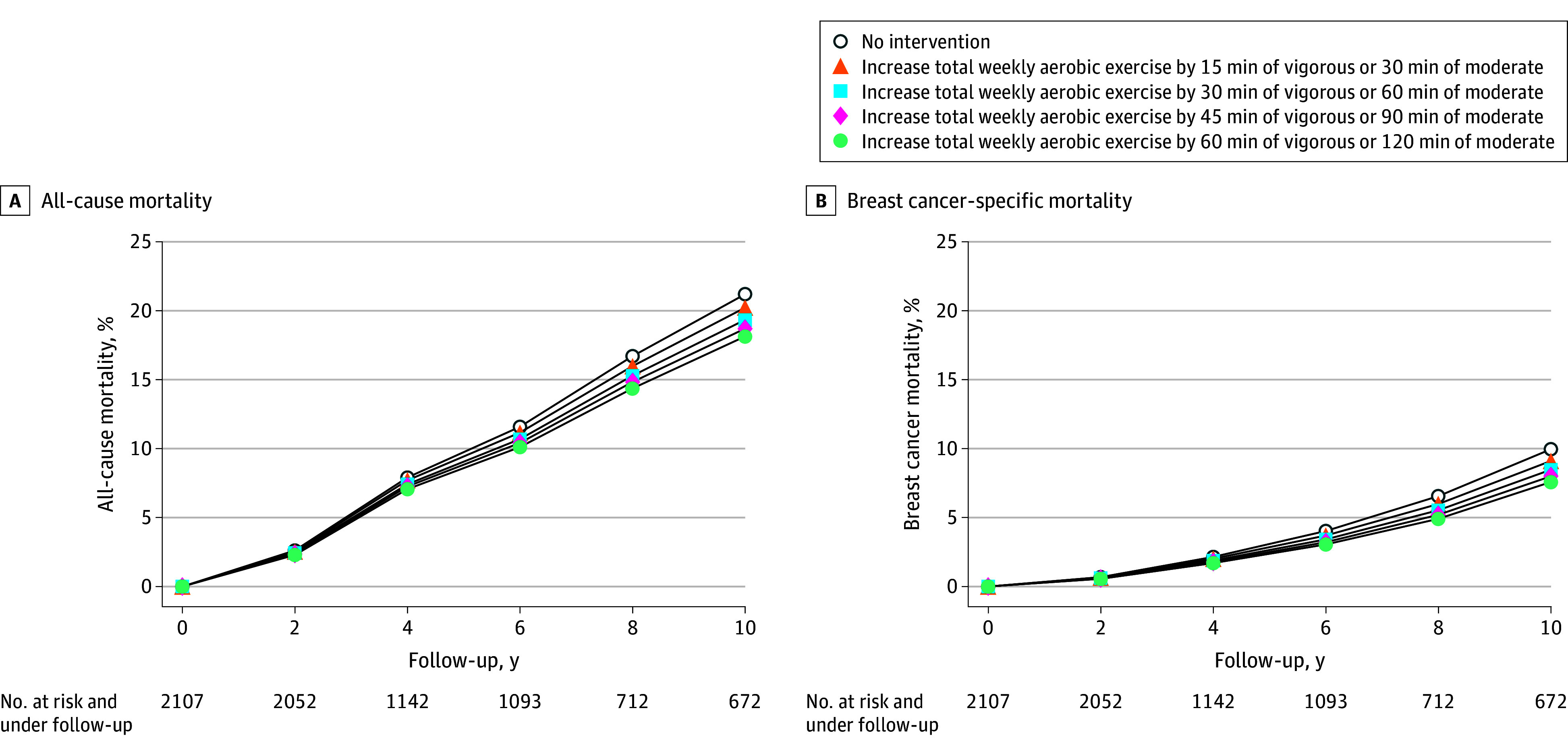
Estimated Risk Curves for All-Cause and Breast Cancer–Specific Mortality Under Different Exercise Strategies in the Second Target Trial Emulation, Pathways Study (2005-2021) Strategies are tailored based on evolving individual characteristics. That is, individuals are no longer required to increase their aerobic exercise levels if and when they develop myocardial infarction, stroke, congestive heart failure, distant recurrence, or swelling that interferes with exercise.

During the 10-year follow-up, 321 deaths occurred (eTable 5 in Supplement 1). The observed risk of all-cause mortality was 21.9%. Using the parametric g-formula, the estimated 10-year risk of all-cause mortality under no intervention was 21.2% (95% CI, 18.7%-23.2%). The estimated 10-year all-cause mortality risks under different tailored exercise strategies ranged from 18.1% (95% CI, 15.5%-20.2%) to 20.2% (95% CI, 17.9%-22.1%). The proportion of individuals who would need to increase their exercise levels under these strategies ranged from 88.6% to 100%. Tailored strategies requiring increases in total aerobic exercise were associated with lower all-cause mortality (Figure 2 and Table 3; eFigure 3 in Supplement 1). Compared with no intervention, a tailored strategy requiring women to increase their total weekly aerobic exercise by 60 minutes of vigorous or 120 minutes of moderate exercise was associated with a 3.1 (95% CI, 2.0-4.6)–percentage point lower 10-year risk of all-cause mortality.

**Table 3.** Estimated 10-Year Risks of All-Cause and Breast Cancer–Specific Mortality Under Different Tailored Exercise Strategies in the Second Target Trial Emulation, Pathways Study (2005-2021)^a^

Exercise strategy^b^	All-cause mortality			Breast cancer–specific mortality		
	10-y Risk, % (95% CI)	Risk difference, % (95% CI)	Risk ratio (95% CI)	10-y Risk, % (95% CI)	Risk difference, % (95% CI)	Risk ratio (95% CI)
No intervention	21.2 (18.7 to 23.2)	0 [Reference]	1.0 [Reference]	10.0 (8.0 to 11.5)	0 [Reference]	1.0 [Reference]
Increase total weekly aerobic exercise by 15 min of vigorous or 30 min of moderate exercise	20.2 (17.9 to 22.1)	−1.0 (−1.5 to −0.6)	0.95 (0.93 to 0.97)	9.1 (7.5 to 10.7)	−0.9 (−1.2 to −0.4)	0.91 (0.88 to 0.96)
Increase total weekly aerobic exercise by 30 min of vigorous or 60 min of moderate exercise	19.3 (17.0 to 21.2)	−1.9 (−2.7 to −1.1)	0.91 (0.87 to 0.94)	8.5 (6.8 to 10.0)	−1.5 (−2.2 to −0.7)	0.85 (0.79 to 0.92)
Increase total weekly aerobic exercise by 45 min of vigorous or 90 min of moderate exercise	18.7 (16.3 to 20.6)	−2.5 (−3.8 to −1.6)	0.88 (0.83 to 0.92)	8.0 (6.3 to 9.5)	−2.0 (−2.9 to −1.0)	0.80 (0.72 to 0.89)
Increase total weekly aerobic exercise by 60 min of vigorous or 120 min of moderate exercise	18.1 (15.5 to 20.2)	−3.1 (−4.6 to −2.0)	0.86 (0.78 to 0.90)	7.6 (5.9 to 9.2)	−2.4 (−3.5 to −1.2)	0.76 (0.66 to 0.87)

^a^ Estimates were obtained using the parametric g-formula, which included baseline covariates (age, race and ethnicity, educational attainment, smoking status, menopausal status, weighted Elixhauser Comorbidity Index, cancer stage, nodal status, hormone receptor status, year of diagnosis, initial treatment with hormonal therapy, initial treatment with chemotherapy, initial treatment with radiotherapy, initial surgery type) and time-varying covariates (body mass index, muscle strengthening exercise, total aerobic exercise, and development of myocardial infarction, stroke, congestive heart failure, distant recurrence, or swelling that interferes with exercise).

^b^ Strategies are tailored based on evolving individual characteristics. That is, individuals are no longer required to increase their aerobic exercise levels if and when they develop myocardial infarction, stroke, congestive heart failure, distant recurrence, or swelling that interferes with exercise.

#### Breast Cancer–Specific Mortality

During the 10-year follow-up, 139 deaths due to breast cancer occurred. The observed 10-year risk of breast cancer–specific mortality was 10.6%. Using the parametric g-formula, the estimated 10-year risk of breast cancer–specific mortality under no intervention was 10.0% (95% CI, 8.0%-11.5%). Estimated 10-year breast cancer–specific mortality risks under tailored exercise strategies ranged from 7.6% (95% CI, 5.9%-9.2%) to 9.1% (95% CI, 7.5%-10.7%). Tailored strategies requiring increases in total aerobic exercise were associated with lower breast cancer–specific mortality (Figure 2 and Table 3; eFigure 3 in Supplement 1). Compared with no intervention, a tailored strategy requiring women to increase their total weekly aerobic exercise by 60 minutes of vigorous or 120 minutes of moderate exercise was associated with a 2.4 (95% CI, 1.2-3.5)–percentage point lower 10-year risk of breast cancer–specific mortality. When we sequentially applied each extension, estimates changed the most when eligible women could be diagnosed with stage I breast cancer (8-year all-cause mortality risk for the recreational aerobic exercise strategy was 6.1 [95% CI, 3.3-9.0] percentage points lower than a strategy based on the health education strategy of the CHALLENGE trial) (eTable 6 in Supplement 1) and when the strategies required incremental increases in exercise (eg, 8-year all-cause mortality for an increase of 60 minutes of vigorous or 120 minutes of moderate exercise per week was 2.7 [95% CI, 1.6-3.6] percentage points lower than for no intervention) (eTable 6 in Supplement 1).

#### Sensitivity Analyses

Estimates for the negative outcome control of questionnaire nonresponse were null (eTable 7 in Supplement 1). Results were similar in other sensitivity analyses (eTable 8 and eFigure 4 in Supplement 1).

#### Other Analyses

Estimates were smaller among women with any baseline level of exercise (eg, 10-year all-cause mortality risk for an increase of 60 minutes of vigorous or 120 minutes of moderate exercise per week was 1.5 [95% CI, 0.9-2.5] percentage points lower than for no intervention) (eTable 9 in Supplement 1). Estimates were larger under strategies requiring women to be insufficiently active, active, or highly active compared with minimally active (eg, 10-year all-cause mortality risk for highly active was 16.2 [95% CI, 7.4-24.6] percentage points lower than for minimally active) (eTable 10 in Supplement 1).

## Discussion

We used observational data to emulate a target trial that approximately replicated the results of the CHALLENGE trial^3^ among breast cancer survivors. Next, we extended these findings to consider the effects of more pragmatic, tailored exercise strategies in a second target trial that was emulated in a broader population. We found that both all-cause and breast cancer–specific mortality risks were lower under tailored strategies that required women to increase their exercise levels only until they were diagnosed with a potentially serious condition. Estimated mortality risks were lowest under a strategy that required increases of 60 minutes of vigorous or 120 minutes of moderate exercise per week. However, meaningful risk reductions were also observed for strategies that required more modest increases in exercise.

These findings are relevant for the clinical management of breast cancer because documentation of exercise recommendations by a medical oncologist is now required for the national accreditation of breast cancer centers in the US.^33^ However, less than 40% of US health care professionals report having discussed exercise with cancer survivors,^34^ in part due to lack of confidence in current research.^35,36^ Previous research^37^ also suggests that clinicians need to be better equipped to prescribe tailored exercise strategies to breast cancer survivors. Our findings could potentially support such discussions.

Although no randomized trial has evaluated the long-term effects of exercise on mortality among breast cancer survivors, we used observational data to replicate the protective estimates of the CHALLENGE trial^3^ among individuals newly diagnosed with breast cancer. These observational findings are important because, although randomized trials are the preferred option, they are difficult to conduct. For example, the CHALLENGE trial took 15 years to recruit 889 patients across 6 countries. Trials also often recruit a select group of cancer survivors (eg, individuals with stage II or III disease, as in the CHALLENGE trial,^3^ or individuals with obesity, as in the Breast Cancer Weight Loss [BWEL] trial).^38^ In contrast, we included a broad range of women, including women with stage I disease, who represent a large proportion of the breast cancer survivors in the US.^39^ We were also able to estimate the effects of 5 different exercise strategies, sustained for up to 8 years, on 10-year mortality risks. Although it would be logistically challenging to estimate these effects in a randomized trial, our results highlight the need for a trial to confirm these findings.

Several prior observational studies^30,40,41,42,43^ have also reported that high levels of exercise are associated with lower mortality among breast cancer survivors. However, with few exceptions,^30,43^ estimates from prior observational studies^40,41,42^ are difficult to interpret because the analyses do not correspond to interventions that are currently used. For example, many prior studies^40,41,42^ have relied on exercise categories that cannot be mapped to realistic recommendations for breast cancer survivors because the categories often imply hypothetical interventions that would require active, healthy women to decrease their exercise. When we emulated similar strategies, we found estimates that were larger than those reported in the CHALLENGE trial (eTable 10 in Supplement 1). Furthermore, prior studies frequently relied on a single measurement of exercise (which precludes the assessment of sustained exercise behaviors), assessed associations with prediagnostic exercise (which does not correspond to an actionable intervention because an individual with cancer cannot change their exercise before diagnosis), or did not consider strategies that are realistically tailored to evolving characteristics (which can introduce substantial bias if, as is expected, some individuals are unable to continuously sustain high levels of exercise due to comorbidities or other characteristics).^43^

In contrast to these prior observational studies, we specified the protocols of 2 target trials with realistic, tailored exercise strategies that were sustained over time^43^ and then emulated them using high-quality observational data. We leveraged a unique dataset that combined the strengths of electronic health records and questionnaire data to enhance the ascertainment of both clinical and exercise variables. Our target trial approach avoided common design-related biases and improved the interpretability of the estimates because they can be mapped to an intervention that can actually be implemented. We also used the parametric g-formula, a method that appropriately accounts for time-varying confounders that may be affected by prior exercise, facilitates the incorporation of competing events within a causal framework, and allows for the emulation of strategies that are tailored based on time-varying characteristics. Our results using these approaches were compatible with 2 previous studies^30,43^ applying a similar causal inference framework to evaluate the effects of hypothetical interventions on exercise and other lifestyle factors. However, in contrast to these prior studies,^30,43^ we began by approximately replicating the results of an existing randomized trial, which increased our confidence in the validity of the observational data. Furthermore, the strategies we emulated in the second target trial required incremental, individualized increases in exercise levels above a woman’s current levels, which may be an attainable goal for breast cancer survivors.

### Limitations

Although our study has several strengths, it also has limitations. First, as in any observational analysis, we cannot rule out potential unmeasured confounding. For example, there could be unmeasured confounding due to other lifestyle factors (eg, sleep and alcohol use) or if women who were less likely to die were also more likely to engage in high levels of exercise (sometimes referred to as healthy adherer bias). However, we used several approaches to allay potential confounding concerns^44^: (1) we approximately replicated the results of a randomized trial in a different population, (2) we specified strategies that realistically allowed women to engage in as much exercise as they were able after the diagnosis of a serious condition, and (3) we carefully adjusted for a broad array of potential baseline and time-varying confounders, including demographic factors such as educational attainment, race, and ethnicity. We also illustrated similar results across several sensitivity analyses, including analyses that can be unbiased in the presence of unmeasured confounding.^27^ Second, we were unable to replicate every aspect of the CHALLENGE trial due to irreconcilable differences between the observational data and the trial, which precluded a more formal benchmarking effort.^45^ For example, the CHALLENGE trial was restricted to individuals who had recently completed adjuvant chemotherapy, which we were unable to replicate with the available observational data. Third, we cannot rule out other potential biases, such as measurement error, selection bias, or model misspecification. However, we used previously validated exercise data^12^ and adjusted for potential selection bias due to loss to follow-up using the parametric g-formula and a rich set of covariates. Our results were also similar in multiple sensitivity analyses assessing these biases. Fourth, because women only reported exercise after diagnosis, we were unable to directly adjust for prediagnostic exercise. Nevertheless, in an auxiliary dataset with prediagnostic data, results were similar with vs without adjustment for prediagnostic behaviors.^43^ Fifth, our strategies focused on increases in individual exercise levels. However, achieving and sustaining these individual increases may require changes in clinical, social, and environmental factors. Sixth, because we used as a comparator in the second target trial the risk under usual exercise among women with low baseline exercise levels in the Pathways Study (ie, no intervention) and specified strategies that required women to increase their exercise beyond what they were already doing, our estimates may not be transportable to populations with different exercise patterns or baseline risks of mortality. Specifically, women in the Pathways Study represent an insured population receiving care through an integrated health care system in California, which does not reflect the experience of all breast cancer survivors in the US.^46,47^

## Conclusions

In this cohort study with a target trial emulation design, we used observational data to replicate the results of a randomized trial in a new population and then estimated meaningful decreases in mortality under more pragmatic, tailored exercise strategies that required women diagnosed with stage I to III breast cancer to increase their exercise levels. These findings could help inform decisions about tailored exercise strategies that are adapted to evolving individual characteristics.

## Notes

Supplement 1. **eMethods 1.** Supplemental methods **eTable 1.** Summary of the CHALLENGE randomized trial, specification of the target trial that approximately mirrored it, and two target trial emulations **eTable 2.** List of exercise activities included in the Arizona Activity Frequency Questionnaire **eTable 3.** Summary of covariates and models used in the two target trial emulations **eTable 4.** Summary of sensitivity analyses implemented in the second target trial emulation **eTable 5.** Number of individuals who were at risk, died and censored by follow-up interval in the first and second target trial emulations **eTable 6.** Sequential application of extensions to target trial 1 **eTable 7.** Sensitivity analysis changing the outcome to questionnaire non-responses (negative outcome control) **eTable 8.** Other sensitivity analyses **eTable 9.** Estimates among women with any baseline level of exercise **eTable 10.** Strategies requiring women to be insufficiently active, active, or highly active, as compared with minimally active **eFigure 1.** Flowchart of eligible individuals for the first target trial emulation **eFigure 2.** Flowchart of eligible individuals for the second target trial emulation **eFigure 3.** 10-year risk differences for all-cause and breast cancer-specific mortality comparing increases in aerobic exercise vs. no increase (i.e. usual aerobic exercise) in the second target trial emulation **eFigure 4.** Comparison of inverse probability weighted vs. parametric g-formula estimates of means of the time-varying covariates under no intervention (i.e., usual exercise levels) in the second target trial emulation **eMethods 2.** TARGET checklist
eReferences

Supplement 2.
Data Sharing Statement
