# Supplementary material for: Tailored Exercise Strategies and Mortality Among Breast Cancer Survivors
Source: JAMA Netw Open. 2026 Apr 13;9(4):e265177. doi: 10.1001/jamanetworkopen.2026.5177 (PMC13077521; doi:10.1001/jamanetworkopen.2026.5177)
Supplement: Supplement 1. — eMethods 1. Supplemental methods eTable 1. Summary of the CHALLENGE randomized trial, specification of the target trial that approximately mirrored it, and two target trial emulations eTable 2. List of exercise activities included in the Arizona Activity Frequency Questionnaire eTable 3. Summary of covariates and models used in the two target trial emulations eTable 4. Summary of sensitivity analyses implemented in the second target trial emulation eTable 5. Number of individuals who were at risk, died and censored by follow-up interval in the first and second target trial emulations eTable 6. Sequential application of extensions to target trial 1 eTable 7. Sensitivity analysis changing the outcome to questionnaire non-responses (negative outcome control) eTable 8. Other sensitivity analyses eTable 9. Estimates among women with any baseline level of exercise eTable 10. Strategies requiring women to be insufficiently active, active, or highly active, as compared with minimally active eFigure 1. Flowchart of eligible individuals for the first target trial emulation eFigure 2. Flowchart of eligible individuals for the second target trial emulation eFigure 3. 10-year risk differences for all-cause and breast cancer-specific mortality comparing increases in aerobic exercise vs. no increase (i.e. usual aerobic exercise) in the second target trial emulation eFigure 4. Comparison of inverse probability weighted vs. parametric g-formula estimates of means of the time-varying covariates under no intervention (i.e., usual exercise levels) in the second target trial emulation eMethods 2. TARGET checklist eReferences [file jamanetwopen-e265177-s001.pdf]

Supplemental Online Content

Jayasekera J, Ergas IJ, Schneider J, et al. Tailored exercise strategies and mortality among breast cancer survivors. *JAMA Netw Open*. 2026;9(4):e265177. doi:10.1001/jamanetworkopen.2026.5177

eMethods 1. Supplemental methods
eTable 1. Summary of the CHALLENGE randomized trial, specification of the target trial that approximately mirrored it, and two target trial emulations
eTable 2. List of exercise activities included in the Arizona Activity Frequency Questionnaire
eTable 3. Summary of covariates and models used in the two target trial emulations
eTable 4. Summary of sensitivity analyses implemented in the second target trial emulation
eTable 5. Number of individuals who were at risk, died and censored by follow-up interval in the first and second target trial emulations
eTable 6. Sequential application of extensions to target trial 1
eTable 7. Sensitivity analysis changing the outcome to questionnaire non-responses (negative outcome control)
eTable 8. Other sensitivity analyses
eTable 9. Estimates among women with any baseline level of exercise
eTable 10. Strategies requiring women to be insufficiently active, active, or highly active, as compared with minimally active
eFigure 1. Flowchart of eligible individuals for the first target trial emulation
eFigure 2. Flowchart of eligible individuals for the second target trial emulation
eFigure 3. 10-year risk differences for all-cause and breast cancer-specific mortality comparing increases in aerobic exercise vs. no increase (ie, usual aerobic exercise) in the second target trial emulation
eFigure 4. Comparison of inverse probability weighted vs. parametric g-formula estimates of means of the time-varying covariates under no intervention (i.e., usual exercise levels) in the second target trial emulation
eMethods 2. TARGET checklist
eReferences

This supplemental material has been provided by the authors to give readers additional information about their work.

© 2026 Jayasekera J et al. *JAMA Network Open*.

eMethods 1. Supplemental methods

DETAILS OF THE PARAMETRIC G-FORMULA

Below we provide technical details of the analytic approach employed in our two target trial emulations. We begin by defining relevant notation.

Notation

Let k represent each 2-year interval of follow-up during which study variables are measured for each of the $i = 1, 2, \ldots n$ eligible individuals. Assume individuals are independent and identically distributed, and thus the i subscript can be suppressed.

Let Y_k represent the development of the outcome of all-cause mortality by interval k ($Y_k = 1$ if died by interval k, $Y_k = 0$ otherwise), C_k represent censoring due to loss to follow-up as a result of questionnaire non-response by interval k ($C_k = 1$ if lost to follow-up by interval k, $C_k = 0$ otherwise), A_k represent total aerobic exercise in interval k, and L_k represent a vector of measured covariates at the start of interval k (e.g., cancer stage, hormone receptor status, etc.). Furthermore, let A_k^* represent the natural value of aerobic exercise in interval k, defined as the aerobic exercise that would have been observed at interval k had the intervention been discontinued right before k, and let g represent a tailored exercise strategy. For illustration, throughout this section we consider the strategy g that is defined as follows:

> "*Increase total weekly aerobic exercise by 60 minutes vigorous or 120 minutes moderate*: Engage in an additional 60 minutes of vigorous aerobic exercise / week or 120 minutes of moderate aerobic exercise / week or an equivalent combination of vigorous and moderate aerobic exercise (400 MET-minutes / week). Strategies are sustained for 8 years. Individuals can choose the type, frequency, intensity, and duration of aerobic exercise. The intervention is discontinued if and when an individual develops myocardial infarction, stroke, congestive heart failure, distant recurrence, or swelling that interferes with exercise. During the intervention period, participants must respond to follow-up questionnaires administered approximately every 2 years to update information on exercise and other prognostic factors."

We use overbars to denote the history of a variable through a specified time point and lower-case letters to denote possible realizations of a random variable (e.g., l_k is a possible realization of L_k). For notational simplicity, we assume each variable is binary and use summations, but when $(\bar{A}_k, \bar{L}_k)$ contains continuous variables, these summations can be replaced by integrals.

Extended parametric g-formula

© 2026 Jayasekera J et al. *JAMA Network Open.*

We used the extended parametric g-formula to estimate the counterfactual risk of the outcome of all-cause mortality by interval $k+1$ had everyone followed strategy g and had no one been lost to follow-up, $\Pr[Y_{k+1}^{g,\bar{c}=0}=1]$.[1] This approach is a form of standardization, where the conditional hazard under strategy g is standardized to the joint distribution of the time-varying covariates. For the intervention density $f^{d}(a_k|a_k^{*},\bar{l}_k,\bar{a}_{k-1},\overline{C_k}=\bar{Y}_k=0)$ that specifies an increase in total weekly aerobic exercise by 60 minutes vigorous or 120 minutes moderate, the extended parametric g-formula is defined as:

$$\Pr[Y_{k+1}^{g,\bar{c}=0}=1]=\sum_{\bar{a}_K}\sum_{\bar{a^*}_K}\sum_{\bar{l}_K}\sum_{k=0}^{K}\Pr\left[Y_{k+1}=1\middle|\bar{L}_k=\bar{l}_k,\bar{A}_k=\bar{a}_k,\bar{C}_{k+1}=\bar{Y}_k=0\right]\times$$
$$\prod_{j=0}^{k}\{\Pr[Y_j=0|\bar{L}_{j-1}=\bar{l}_{j-1},\bar{A}_{j-1}=\bar{a}_{j-1},\bar{C}_j=\bar{Y}_{j-1}=0]\times$$
$$f^{d}\left(a_j\middle|a_j^{*},\bar{l}_j,\bar{a}_{j-1},\bar{C}_j=\bar{Y}_j=0\right)\times f\left(a_j^{*}\middle|\bar{l}_j,\bar{a}_{j-1},\bar{C}_j=\bar{Y}_j=0\right)\times$$
$$f\left(l_j\middle|\bar{l}_{j-1},\bar{a}_{j-1},\bar{C}_j=\bar{Y}_j=0\right)\}$$

(Equation 1)

Where $f\left(a_k^{*}\middle|\bar{l}_k,\bar{a}_{k-1},\overline{C_k}=\bar{Y}_k=0\right)$ is the observed aerobic exercise density conditional on the measured covariate history, evaluated at some possibly realized values $(\bar{A}_k,\bar{L}_k)=(a_k^{*},\ \bar{a}_{k-1},\bar{l}_k)$.

Due to the high-dimensionality of our data, we estimated equation (1) parametrically. To do this, we first fit parametric pooled over time models for each component density, including:

i) the conditional densities of the time-varying covariates conditional on past covariate history
ii) the conditional discrete hazard of the outcome conditional on past covariate history

The covariates and modeling approaches for this step are described in eTable 3. We then used Monte Carlo methods to generate counterfactual outcome distributions for the entire population had everyone followed strategy g and not been lost to follow-up, which was implemented in the following steps:

i) sample with replacement from the study population to generate a 10,000 person population
ii) at baseline ($k=0$), set confounder values l_0 and natural aerobic exercise values a_0^{*} to their observed values
iii) during follow-up ($k>0$), predict the time-varying covariates for each interval k based on the estimated model coefficients from the parametric pooled over time models described above and previously generated covariate values through $k-1$ under strategy g

© 2026 Jayasekera J et al. *JAMA Network Open.*

iv) at each interval $k = 0, 1, ... K$, set interval k aerobic exercise to the value it would take under strategy g
v) predict for each follow-up time from $k = 1,2, ... K + 1$ the conditional discrete hazard of the outcome given past covariates that were generated under strategy g
vi) calculate the cumulative probability of the outcome by interval $k + 1$, using the predicted hazards generated in the last step
vii) standardize the probabilities of the outcome obtained in the last step by taking the average over all generated person-histories.

This process was repeated to generate risks under each tailored exercise strategy of interest. Nonparametric bootstrapping with 500 resamples was used to obtain percentile-based 95% confidence intervals for the estimated risks, risk differences, and risk ratios. For comparison with the CHALLENGE trial, hazard ratios were also estimated via Cox proportional hazards models.

ASSUMPTIONS

Our primary aim was to estimate observational analogues of per-protocol effects using the extended parametric g-formula, as described above. Valid estimation of these per-protocol effects (whether in a randomized target trial or its observational emulation) requires several assumptions. First, under the (non-extended) parametric g-formula, we require the assumptions of sequential positivity, consistency, exchangeability (including no unmeasured confounding, selection bias, or measurement error), and no model misspecification. Under the extended parametric g-formula and in the setting of data that is only collected at intervals approximately every 2 years, we need to make additional assumptions, as described elsewhere.[2,3]

LOSS TO FOLLOW-UP

Individuals were considered lost to follow-up if they did not return a questionnaire in a given follow-up interval.

MISSING DATA

Eligible individuals were required to have no missing data at baseline.

Throughout the follow-up, if data on specific covariate(s) were missing because those variables were not ascertained on a given follow-up questionnaire, then models for these covariates excluded those follow-up intervals. Modeled estimates were then obtained by carrying forward, for each individual, the last value generated for that covariate by the parametric g-formula under the specified exercise strategies. If data on specific covariate(s) were missing due to item non-response, then we carried forward the last reported values of those covariate(s) until a new value was available.

SENSITIVITY ANALYSES

© 2026 Jayasekera J et al. *JAMA Network Open.*

We conducted several sensitivity analyses to assess the robustness of our target trial emulation results to the underlying assumptions described above.

First, we conducted analyses to evaluate the robustness of our results to the assumption that all prognostic factors that predict exercise patterns throughout the follow-up were correctly adjusted for (i.e., no unmeasured confounding) and that all women who were required to increase their exercise were able to do so (i.e., sequential positivity). These analyses included 1) requiring eligible individuals to have a baseline weighted Elixhauser Comorbidity Index < 2 (to reduce potential unmeasured baseline confounding or positivity violations due to co-morbid conditions), 2) requiring individuals to increase their exercise only until they developed a broader set of serious conditions, which additionally included angina, arrythmia, other cardiovascular conditions, other respiratory conditions, fractures, osteoporosis, and lymphedema (to reduce potential unmeasured time-varying confounding or sequential positivity violations due to these additional conditions), 3) requiring individuals to increase their exercise only until 2 years before one of the original conditions developed (to reduce potential sequential positivity violations or unmeasured confounding due to healthy adherer bias or reverse causation, if people who had symptoms of these conditions before diagnosis were less likely or able to increase their exercise and also more likely to die),[4] 4) changing the outcome to questionnaire non-response (a negative control outcome that we hypothesized would not be directly affected by exercise but could be similarly confounded due to disease severity or health-seeking behavior),[5] and 5) additionally adjusting for baseline income and HER2 status (to mitigate potential residual confounding due to socioeconomic factors and tumor characteristics).

We also conducted several analyses to assess the sensitivity of our findings to potential model misspecification, including 1) changing the arbitrary order in which time-varying covariates collected within the same questionnaire cycle were modeled within the parametric g-formula and 2) comparing means of the time-varying covariates under no intervention estimated using two different modeling approaches (the parametric g-formula and inverse probability weighting), as described previously.[6]

Details on these analyses are provided in eTable 4. In addition to the above analyses, we also 1) adjusted for potential time-varying selection bias due to loss to follow-up in all analyses, 2) relied on previously validated questionnaire data and rigorously collected electronic health records to minimize potential measurement error, and 3) assumed that different types of moderate or vigorous exercise would have similar effects on mortality, as part of the consistency assumption.

OTHER ANALYSES

We conducted several other analyses.

First, to evaluate the effects of the tailored strategies requiring increases in exercise levels among women with any baseline level of exercise, we emulated a target trial which was identical to the second target trial described in the main text, except that eligible individuals

© 2026 Jayasekera J et al. *JAMA Network Open.*

would not have to be currently engaging in less than the equivalent of 150 minutes per week of moderate-to-vigorous intensity aerobic exercise.

Second, to compare our results to findings from prior observational studies which often (i) did not consider tailored strategies, (ii) imposed lower/upper limits on exercise (e.g., engage in 75 – 150 minutes of vigorous aerobic exercise/week), and (iii) estimated effects in comparison to minimal exercise levels, we emulated another target trial which was identical to the second target trial described in the main text, except that individuals would be assigned to one of the following strategies:

1) *Minimally active*: Engage in < 15 minutes of vigorous aerobic exercise / week or < 30 minutes of moderate aerobic exercise / week or an equivalent combination of vigorous and moderate aerobic exercise (< 100 MET-minutes / week).
2) *Insufficiently active*: Engage in 15 – < 75 minutes of vigorous aerobic exercise / week or 30 – < 150 minutes of moderate aerobic exercise / week or an equivalent combination of vigorous and moderate aerobic exercise (100 – < 500 MET-minutes / week).
3) *Active*: Engage in 75 – < 150 minutes of vigorous aerobic exercise / week or 150 – < 300 minutes of moderate aerobic exercise / week or an equivalent combination of vigorous and moderate aerobic exercise (500 – < 1,000 MET-minutes / week).
4) *Highly active*: Engage in ≥ 150 minutes of vigorous aerobic exercise / week or ≥ 300 minutes of moderate aerobic exercise / week or an equivalent combination of vigorous and moderate aerobic exercise (≥ 1,000 MET-minutes / week).

These strategies are once again sustained for up to 8 years. Individuals are always required to meet the exercise levels described above, even if they develop myocardial infarction, stroke, congestive heart failure, distant recurrence, or swelling that interferes with exercise.

© 2026 Jayasekera J et al. *JAMA Network Open.*

eTable 1. Summary of the CHALLENGE randomized trial,[7] specification of a target trial that approximately mirrored it, and two target trial emulations using observational data from the Pathways Study

Protocol Component	CHALLENGE Randomized Trial	Target Trial Specification	Target Trial Emulation (Target Trial 1)	Target Trial Emulation (Target Trial 2)
Eligibility criteria	• Women and men aged ≥ 18 years<br>• Diagnosed with incident high-risk stage II or stage III adenocarcinoma of the colon between 2009 and 2024<br>• No substantial comorbid condition that could preclude participation in a exercise program as determined by the investigator<br>• Currently engaging in less than the equivalent of 150 minutes per week of moderate-to-vigorous intensity recreational aerobic exercise<br>• No prior history of other invasive cancer, except adequately treated nonmelanoma skin cancer, curatively treated in situ cancer of the cervix, other solid tumors, Hodgkin lymphoma, or non-Hodgkin lymphoma curatively treated with no evidence of disease for > 5 years<br>• Eastern Cooperative Oncology Group (ECOG) performance-status score of 0 or 1<br>• Able to complete at least two stages of submaximal treadmill test (walking at a casual pace for 6 minutes) or the 6-minute walk test<br>• Likely to participate in an exercise program, as assessed by the investigator	• Women aged ≥ 21 years who are members of the Kaiser Permanente Northern California (KPNC) healthcare system; speakers of English, Spanish, Cantonese or Mandarin; and residing within a 65-mile radius of a field interviewer in Northern California<br>• Diagnosed with incident stage II or III invasive breast cancer between 2005 and 2013<br>• No substantial comorbid condition that could preclude participation in an exercise program, defined as a weighted Elixhauser Comorbidity Index ≥ 14 during the 12 months prior to diagnosis or swelling that interfered with exercise within the past 6 months<br>• Currently engaging in less than the equivalent of 150 minutes per week of moderate-to-vigorous intensity recreational aerobic exercise<br>• No prior history of other invasive cancer	Same as for the target trial, except that participants are additionally required to have complete baseline data. Eligibility criteria are ascertained via electronic health record linkage and periodic questionnaires.	Same as for target trial 1, except that:<br>• Women can be diagnosed with incident stage I, II, or III invasive breast cancer<br>• Women can have a prior history of other invasive cancer

© 2026 Jayasekera J et al. *JAMA Network Open.*

Protocol Component	CHALLENGE Randomized Trial	Target Trial Specification	Target Trial Emulation (Target Trial 1)	Target Trial Emulation (Target Trial 2)
	• Able (i.e., sufficiently fluent) and willing to effectively communicate with the exercise consultant affiliated with the originating cancer center<br>• Able (i.e., sufficiently fluent in English or French) and willing to complete the patient-reported outcome questionnaires, social determinants of exercise measurement, health economics, and exercise questionnaires and logs<br>• Able to complete the baseline exercise test<br>• Underwent complete resection and completed adjuvant chemotherapy within the past 2 to 6 months<br>• No prior radiotherapy as a component of treatment for primary tumor<br>• No concurrent treatment with additional chemotherapy or radiation<br>• No concurrent treatment with any medications deemed by the investigator as likely to preclude participation in an exercise program<br>• No concurrent anticancer treatment including chemotherapy, biological, or targeted agents<br>• Completion of chest x-ray or CT, and CT, MRI or ultrasound of abdomen within 60 days of			

© 2026 Jayasekera J et al. *JAMA Network Open.*

Protocol Component	CHALLENGE Randomized Trial	Target Trial Specification	Target Trial Emulation (Target Trial 1)	Target Trial Emulation (Target Trial 2)
	registration with no evidence of metastatic or locally-recurrent colon cancer.<br>• Carcinoembryonic antigen (CEA) ≤ 5 µg/L<br>• Absolute granulocyte count ≥ 1,000/mm³<br>• Platelet count ≥ 100,000/mm³<br>• Hemoglobin ≥ 100 g/L<br>• Serum creatinine ≤ 1.5 times upper limit of normal (ULN)<br>• Total bilirubin ≤ 1.5 times upper limit of normal (ULN)<br>• Alkaline phosphatase < 2.5 times ULN<br>• ALT < 2 times ULN<br>• Not pregnant or planning to become pregnant within the next 3 years<br>• Provided informed consent			
Exercise strategies	1) *Health education intervention*: Receive general health education materials promoting exercise, healthy nutrition, and standard surveillance<br><br>2) *Recreational aerobic exercise intervention:* Receive general health education materials and participate in a structured aerobic exercise intervention (comprised of behavioral-support sessions and supervised exercise sessions) aimed at increasing weekly	*1) Health education intervention*: Do not let recreational aerobic exercise levels exceed the average levels achieved under the health education intervention of the CHALLENGE trial (~900 MET-minutes / week)<br><br>*2) Recreational aerobic exercise intervention:* Increase weekly aerobic exercise to at least meet the average levels achieved under the recreational	Same as for the target trial. Exercise levels are self-reported using the Arizona Activity Frequency Questionnaire (eTable 2).	*1) No intervention:* Usual aerobic exercise levels<br><br>*2) Increase total weekly aerobic exercise by 15 minutes vigorous or 30 minutes moderate*: Engage in an additional 15 minutes of vigorous aerobic exercise / week or 30 minutes of moderate aerobic exercise / week or an equivalent combination of vigorous and moderate

Protocol Component	CHALLENGE Randomized Trial	Target Trial Specification	Target Trial Emulation (Target Trial 1)	Target Trial Emulation (Target Trial 2)
	recreational aerobic exercise by at least 10 MET-hours per week<br><br>Both strategies are sustained for 3 years. Individuals can choose the type, frequency, intensity, and duration of aerobic exercise. The intervention is discontinued if and when an individual experiences a disease event, defined as disease recurrence or any new primary cancer	aerobic exercise intervention of the CHALLENGE trial (~1,275 MET-minutes / week)<br><br>Both strategies are sustained for 4 years. Individuals can choose the type, frequency, intensity, and duration of aerobic exercise. The intervention is discontinued if and when an individual experiences a disease event, defined as disease recurrence or second primary breast cancer.<br><br>During the intervention period, participants must respond to follow-up questionnaires administered approximately every 2 years to update information on exercise and other prognostic factors.		aerobic exercise (100 MET-minutes / week).<br><br>*3) Increase total weekly aerobic exercise by 30 minutes vigorous or 60 minutes moderate*: Engage in an additional 30 minutes of vigorous aerobic exercise / week or 60 minutes of moderate aerobic exercise / week or an equivalent combination of vigorous and moderate aerobic exercise (200 MET-minutes / week).<br><br>*4) Increase total weekly aerobic exercise by 45 minutes vigorous or 90 minutes moderate*: Engage in an additional 45 minutes of vigorous aerobic exercise / week or 90 minutes of moderate aerobic exercise / week or an equivalent combination of vigorous and moderate aerobic exercise (300 MET-minutes / week).<br><br>*5) Increase total weekly aerobic exercise by 60 minutes vigorous or 120 minutes moderate*: Engage in an additional 60 minutes of vigorous aerobic

© 2026 Jayasekera J et al. *JAMA Network Open.*

Protocol Component	CHALLENGE Randomized Trial	Target Trial Specification	Target Trial Emulation (Target Trial 1)	Target Trial Emulation (Target Trial 2)
				exercise / week or 120 minutes of moderate aerobic exercise / week or an equivalent combination of vigorous and moderate aerobic exercise (400 MET-minutes / week).<br><br>All strategies are sustained for 8 years. Individuals can choose the type, frequency, intensity, and duration of aerobic exercise. The intervention is discontinued if and when an individual develops myocardial infarction, stroke, congestive heart failure, distant recurrence, or swelling that interferes with exercise. During the intervention period, participants must respond to follow-up questionnaires administered approximately every 2 years to update information on exercise and other prognostic factors. Exercise levels are self-reported as in target trial 1.

© 2026 Jayasekera J et al. *JAMA Network Open.*

Protocol Component	CHALLENGE Randomized Trial	Target Trial Specification	Target Trial Emulation (Target Trial 1)	Target Trial Emulation (Target Trial 2)
Assignment procedures	Individuals are randomly assigned to a strategy (by center, disease stage, body mass index, and ECOG performance status) and are aware of their assigned strategy.	Same as for the CHALLENGE trial except randomized assignment is not stratified by center, disease stage, body mass index, or ECOG performance status.	Each individual is assigned to all exercise strategies.	Same as for target trial 1.
Outcomes	Primary: Disease-specific mortality (defined as disease recurrence, any new primary cancer, or death)<br><br>Secondary: all-cause mortality *(for additional secondary outcomes see ClinicalTrials.gov number NCT00819208)*	All-cause mortality	Same as for the target trial. Deaths were ascertained from family members, medical records, and KPNC mortality files, which include data from KPNC, the state of California, the Social Security Administration, and the National Death Index.	All-cause and breast cancer-specific mortality. Deaths were ascertained as in target trial 1.
Follow-up	For each eligible individual, follow-up begins at assignment to an exercise strategy (baseline) and continues until the outcome of interest, 10 years, or administrative end of follow-up (August 29, 2024), whichever occurs first.	For each eligible individual, follow-up begins at assignment to an exercise strategy (baseline) and continues until the outcome of interest, loss to follow-up (defined as follow-up questionnaire non-response), 10 years, or administrative end of follow-up (December 31, 2021), whichever occurs first.	Same as for the target trial. Baseline was defined as the return date of the diagnostic questionnaire.	Same as for target trial 1.
Causal contrasts	Intention-to-treat effect, reported as risk differences and hazard ratios.	Intention-to-treat effect and per-protocol effect, reported as risk differences, risk ratios, and hazard ratios.	Observational analogue of the per-protocol effect.	Same as for target trial 1 except that hazard ratios are not reported. For breast cancer-specific mortality, these effects are contrasts of the marginal risk of breast cancer-

© 2026 Jayasekera J et al. *JAMA Network Open.*

Protocol Component	CHALLENGE Randomized Trial	Target Trial Specification	Target Trial Emulation (Target Trial 1)	Target Trial Emulation (Target Trial 2)
				specific mortality under each exercise strategy (i.e., total effects).
Identifying assumptions	Intention-to-treat effect: Randomized assignment.<br><br>Per-protocol effect: Sequential exchangeability for exercise conditional on time-varying covariates.<br><br>Additional assumptions are discussed in Supplemental Methods I.	Intention-to-treat effect: Randomized assignment and sequential exchangeability for censoring due to loss to follow-up conditional on the baseline and time-varying covariates described in eTable 3.<br><br>Per-protocol effect: Sequential exchangeability for exercise and censoring due to loss to follow-up conditional on the baseline and time-varying covariates described in eTable 3.<br><br>Additional assumptions are discussed in Supplemental Methods I.	Same per-protocol effect identifying assumptions as for the target trial. Covariates were ascertained via electronic health record linkage and periodic questionnaires.	Same as for target trial 1.
Data analysis	Intention-to-treat analysis: 5- and 8-year risks under each strategy are compared using the Kaplan-Meier estimator and Cox proportional hazards models.	Intention-to-treat analysis: Same as for the CHALLENGE trial.<br>Per-protocol analysis: 8-year risks under each strategy are compared using the parametric g-formula, incorporating both baseline and time-varying covariates (eTable 3). Hazard ratios are estimated using a Cox proportional hazards model.	Same per-protocol analysis as for the target trial.	Same per-protocol analysis as for target trial 1 except that risks are estimated over 10 years and hazard ratios are not estimated. Sensitivity analyses are described in Supplemental Methods I and eTable 4.

© 2026 Jayasekera J et al. *JAMA Network Open.*

Protocol Component	CHALLENGE Randomized Trial	Target Trial Specification	Target Trial Emulation (Target Trial 1)	Target Trial Emulation (Target Trial 2)
		Additional details are provided in Supplemental Methods I.		

Abbreviations: MET = Metabolic Equivalent of Task; KPNC = Kaiser Permanente Northern California.

© 2026 Jayasekera J et al. *JAMA Network Open.*

eTable 2. List of exercise activities included in the Arizona Activity Frequency Questionnaire, Pathways Study

**Recreational activity**	**Non-recreational activity**
Aerobic dance or exercise class	Animal grooming
Baseball	Biking for transportation
Biking	Caring for elderly or disabled people
Brisk walking	Caring for infants and toddlers
Calisthenics	Caring for young children (3-5 years old)
Cross country skiing	Carpentry
Flyfishing or hunting	Climbing up stairs
Golf (not using cart)	Dog walking
Golf (using cart)	Grocery shopping
Hiking	Heavy yard work
Horseback riding	Laundry
Jazz, ballet, modern, tap, hip hop, ethnic dance	Lawn mowing (ride on)
Running	Lawn mower (push)
Skiing	Light yard work
Slow walking	Major cleaning
Soccer	Meal preparation
Social, folk dancing	Moving furniture
Stairmaster	Other shopping
Swimming	Painting
Tennis	Routine cleaning
Volleyball	Walking to bus, work, or to do errands
Weightlifting (muscle-strengthening exercise)	
Yoga	

© 2026 Jayasekera J et al. *JAMA Network Open.*

eTable 3. Summary of covariates and models used in the two target trial emulations, Pathways Study

Covariates	Functional form when modeled as:	
	**Dependent variable**	**Independent variable**
**Baseline covariates**		
Age	N/A	6 categories:<br>1. ≤ 39<br>2. 40 – 49<br>3. 50 – 59<br>4. 60 – 69<br>5. 70 – 79<br>6. ≥ 80
Race and ethnicity	N/A	5 categories<br>1. Non-Hispanic White<br>2. Non-Hispanic Black<br>3. Asian/Pacific-Islander<br>4. Hispanic<br>5. American Indian/Alaskan Native
Educational attainment	N/A	4 categories<br>1. High school or less<br>2. Some college<br>3. College graduate<br>4. Post college
Smoking status	N/A	3 categories<br>1. Never<br>2. Former<br>3. Current
Menopausal status	N/A	2 categories<br>1. Premenopausal<br>2. Postmenopausal

© 2026 Jayasekera J et al. *JAMA Network Open.*

eTable 3. Summary of covariates and models used in the two target trial emulations, Pathways Study

Covariates	Functional form when modeled as:	
	**Dependent variable**	**Independent variable**
Elixhauser Comorbidity Index	N/A	4 categories<br>1. < 0<br>2. 0<br>3. 1<br>4. 2+
Cancer stage	N/A	2-3 categories<br>1. AJCC Stage I *(target trial 2 only)*<br>2. AJCC Stage II<br>3. AJCC Stage III
Nodal status	N/A	Binary indicator<br>0. Lymph node negative<br>1. Lymph node positive
Hormone receptor (estrogen receptor [ER] / progesterone receptor [PR]) status	N/A	3 categories<br>1. ER and PR positive<br>2. ER or PR positive<br>3. ER and PR negative
Year of diagnosis	N/A	4 categories<br>1. 2005 – 2006<br>2. 2007 – 2008<br>3. 2009 – 2010<br>4. 2010 – 2013
Initial treatment with chemotherapy[a]	N/A	Binary indicator<br>0. Not treated<br>1. Treated

© 2026 Jayasekera J et al. *JAMA Network Open.*

eTable 3. Summary of covariates and models used in the two target trial emulations, Pathways Study

Covariates	Functional form when modeled as:	
	**Dependent variable**	**Independent variable**
Initial treatment with hormonal therapy[a]	N/A	Binary indicator<br>0. Not treated<br>1. Treated
Initial treatment with radiotherapy[a]	N/A	Binary indicator<br>0. Not treated<br>1. Treated
Initial surgery type	N/A	3 categories<br>1. No surgery<br>2. Lumpectomy<br>3. Mastectomy
**Time-varying covariates**		
Recreational aerobic exercise *(target trial 1)* or Total aerobic exercise *(target trial 2)*	Linear[b]	Restricted cubic spline with 4 knots placed at approximately the 5th, 25th, 75th, and 95th percentiles
Muscle strengthening exercise	Logistic to log linear[c]	5 categories<br>1. < 0.5 MET-hours<br>2. 0.5 – <1 MET-hours<br>3. 1 – <2 MET-hours<br>4. 2 – <5 MET-hours<br>5. ≥ 5 MET-hours
Body mass index (BMI)	Linear on natural log scale[b]	Restricted cubic spline with 4 knots placed at approximately the 5th, 25th, 75th, and 95th percentiles

© 2026 Jayasekera J et al. *JAMA Network Open.*

eTable 3. Summary of covariates and models used in the two target trial emulations, Pathways Study

Covariates	Functional form when modeled as:	
	**Dependent variable**	**Independent variable**
Development of disease recurrence or second primary breast cancer (*target trial 1*) or myocardial infarction, stroke, congestive heart failure, distant recurrence, or swelling that interferes with exercise (*target trial 2*)	Logistic to failure[d]	Binary indicator of diagnosis:<br>0. No<br>1. Yes<br>+ product term between indicator for diagnosis and continuous time since diagnosis (linear term)
Period of follow-up	N/A	5 categories (1 for each 2-year period):<br>1. Period 0 (baseline)<br>2. Period 1<br>3. Period 2<br>4. Period 3<br>5. Period 4

[a]Initial treatment: Initial treatments were defined as treatments received within the first 12 months after breast cancer diagnosis. These variables were incorporated as baseline covariates under the assumption that exercise patterns within the first 12 months of diagnosis would not influence cancer treatment decisions. [b]Linear: Variables predicted by a linear model were assigned a value equal to the predicted value plus the standard error multiplied by a random number from a standard normal distribution (mean 0 and standard deviation 1). Therefore, two individuals with the same covariate history were not necessarily predicted to have exactly the same value at the next time point. Predicted values were truncated so that they did not fall outside of the observed range of the data. [c]Logistic to log linear: Variables with many zero values were predicted in two stages. First, we fit a logistic model for an indicator that the variable was nonzero. Second, we fit a linear model for the natural log of the nonzero values. Predicted values were truncated so that they did not fall outside of the observed range of the data. [d]Logistic to failure: Variables predicted by a logistic model were assigned a value of 1 if the predicted probability was greater than a random number from a uniform distribution. After the first 1 was generated, the value was set to 1 thereafter. Abbreviations: AJCC = American Joint Committee on Cancer; MET = Metabolic Equivalent of Task.

© 2026 Jayasekera J et al. *JAMA Network Open.*

eTable 4. Summary of sensitivity analyses implemented in the second target trial emulation, Pathways Study

Sensitivity analysis	Description of modification(s) to the target trial	Primary rationale	N eligible	N deaths	N deaths due to breast cancer
**Modifications to eligibility criteria**					
Require eligible individuals to have a baseline weighted Elixhauser Comorbidity Index < 2	*Eligibility criteria*: Eligible individuals are required to have a baseline weighted Elixhauser Comorbidity Index < 2	Further restricting the target population based on Elixhauser Comorbidity Index may reduce potential unmeasured baseline confounding due to co-morbid conditions.	1,707	221	75
**Modifications to strategies**					
Require individuals to increase exercise until they develop a broader set of serious conditions	*Exercise strategies*: Individuals are no longer required to increase their exercise levels beyond their usual levels if and when they develop myocardial infarction, stroke, congestive heart failure, distant recurrence, swelling that interferes with exercise, angina, arrythmia, other cardiovascular conditions, other respiratory conditions, fractures, osteoporosis, or lymphedema.	Unmeasured confounding or positivity violations may occur among individuals with this broader set of serious conditions. Modifying the strategies such that once an individual develops one of these conditions they no longer have to increase their aerobic exercise levels means that we can obtain unbiased results even if there is unmeasured confounding or positivity violations *after* one of these diagnoses occurs.	2,107	321	139
Require individuals to increase exercise	*Exercise strategies*: Individuals are no longer	Unmeasured confounding (e.g., due to healthy adherer bias or	2,052	266	124

eTable 4. Summary of sensitivity analyses implemented in the second target trial emulation, Pathways Study

Sensitivity analysis	Description of modification(s) to the target trial	Primary rationale	N eligible	N deaths	N deaths due to breast cancer
until 2 years before they develop a serious condition	required to increase their exercise levels beyond their usual levels until 2 years before they develop myocardial infarction, stroke, congestive heart failure, distant recurrence, or swelling that interferes with exercise	reverse causation) or positivity violations may be more likely among individuals who are experiencing symptoms of subclinical disease 2 years before a serious condition is diagnosed. Modifying the strategies such that an individual is no longer required to increase their aerobic exercise levels 2 years prior to the diagnosis of one of these conditions means that we can obtain unbiased estimates even if there is unmeasured confounding or positivity violations up to *two years before* one of these conditions occurs. Note that, although these strategies cannot be directly implemented in the real world (because it is generally unknown who will develop a serious condition in 2 years), these analyses would be unaffected by positivity violations or unmeasured confounding due to healthy adherer bias or reverse causation if 1) exercise only affected these conditions and mortality after 2 years, 2) diagnosed serious conditions were in a preclinical			

© 2026 Jayasekera J et al. *JAMA Network Open*.

eTable 4. Summary of sensitivity analyses implemented in the second target trial emulation, Pathways Study

Sensitivity analysis	Description of modification(s) to the target trial	Primary rationale	N eligible	N deaths	N deaths due to breast cancer
		state for 2 years prior to diagnosis, and 3) these preclinical serious conditions would have been diagnosed or led to death within 2 years.			
**Modifications to outcome**					
Change the outcome to questionnaire non-response	*Outcome:* Questionnaire non-response.	A non-null effect estimate for the negative control outcome of questionnaire non-response may indicate potential unmeasured confounding (e.g., due to cancer disease severity or frailty).	2,107 (1,024 questionnaire non-responses)	NA	NA
**Modifications to adjustment covariates**					
Additionally adjust for baseline income and HER2 status	*Strategy assignment:* Same as for the target trial, except that we assumed that assignment was as if randomized conditional on the following baseline covariates: age, race and ethnicity, educational attainment, smoking status, menopausal status, weighted Elixhauser Comorbidity Index, cancer stage, nodal status,	Additionally adjusting for these variables may reduce potential residual confounding due to socioeconomic factors or tumor characteristics	1,769	262	108

© 2026 Jayasekera J et al. *JAMA Network Open.*

eTable 4. Summary of sensitivity analyses implemented in the second target trial emulation, Pathways Study

Sensitivity analysis	Description of modification(s) to the target trial	Primary rationale	N eligible	N deaths	N deaths due to breast cancer
	hormone receptor status, year of diagnosis, initial treatment with hormonal therapy, initial treatment with chemotherapy, initial treatment with radiotherapy, initial surgery type, income, and HER2 status.				
**Modifications to modeling assumptions**					
Change the order in which time-varying covariates are modeled	*Statistical analysis*: In the main analysis, time-varying covariates were modeled according to the following temporal ordering within each follow-up interval: 1) development of myocardial infarction, stroke, congestive heart failure, distant recurrence, or swelling that interferes with exercise, 2) body mass index, 3) muscle strengthening exercise, 4) aerobic exercise.<br><br>In this sensitivity analysis, we changed this temporal	The parametric g-formula approach we used relied on sequential modeling of covariates, which required specifying an (arbitrary) temporal ordering of covariates that were ascertained form the same follow-up questionnaire. Similar results when altering this arbitrary ordering suggests that results are not substantially influenced by this modeling assumption.	2,107	321	139

© 2026 Jayasekera J et al. *JAMA Network Open*.

eTable 4. Summary of sensitivity analyses implemented in the second target trial emulation, Pathways Study

Sensitivity analysis	Description of modification(s) to the target trial	Primary rationale	N eligible	N deaths	N deaths due to breast cancer
	ordering to: 1) muscle strengthening exercise, 2) development of myocardial infarction, stroke, congestive heart failure, distant recurrence, or swelling that interferes with exercise, 3) body mass index, 4) aerobic exercise.				

eTable 5. Number of individuals who were at risk, died and censored by follow-up interval in the first and second target trial emulations using observational data from the Pathways Study (2005 – 2021)

Interval of follow-up	Number of individuals alive and uncensored due to loss to follow-up at the start of the interval	Number of individuals who died by the end of the interval	Number of individuals who were censored due to questionnaire non-response by the end of the interval	Number of individuals who were censored on December 31, 2021 or 10 years after baseline
**Target Trial 1**				
0 – 2 years	2,107	55	0	0
2 – 4 years	2,052	110	800	0
4 – 6 years	1,142	49	0	0
6 – 8 years	1,093	67	224	90
8 – 10 years	712	40	0	672
**Target Trial 2**				
0 – 2 years	959	42	0	0
2 – 4 years	917	79	372	0
4 – 6 years	466	31	0	0
6 – 8 years	435	31	95	40

© 2026 Jayasekera J et al. *JAMA Network Open*.

eTable 6. Sequential application of extensions to target trial 1 - Estimated 8-year risks of all-cause mortality under different tailored exercise strategies using observational data from the Pathways Study (2005 – 2021)[a]

Exercise strategy	8-year Risk, % (95% CI)	Risk difference, percentage points (95% CI)	Risk ratio (95% CI)
**Target trial 1 (N = 959)**			
Health education intervention[b]	23.8 (20.0, 27.0)	Reference	Reference
Recreational aerobic exercise intervention[b]	15.8 (9.6, 21.3)	-8.0 (-13.3, -3.4)	0.66 (0.42, 0.86)
**Extension – Allow eligible women to be diagnosed with stage I cancer (N = 2,048)**			
Health education intervention[b]	15.6 (13.7, 17.9)	Reference	Reference
Recreational aerobic exercise intervention[b]	9.5 (6.6, 13.0)	-6.1 (-9.0, -3.3)	0.61 (0.43, 0.79)
**Extension – Additionally allow eligible women to have a prior history of cancer (N = 2,107)**			
Health education intervention[b]	16.1 (13.9, 17.9)	Reference	Reference
Recreational aerobic exercise intervention[b]	9.6 (6.3, 12.6)	-6.4 (-9.3, -4.2)	0.60 (0.41, 0.74)
**Extension – Additionally change the exercise strategies (N = 2,107)**			
No intervention	16.7 (14.5, 18.3)	Reference	Reference
Increase total weekly aerobic exercise by 15 minutes vigorous or 30 minutes moderate	15.8 (13.7, 17.6)	-0.9 (-1.2, -0.4)	0.95 (0.93, 0.97)
Increase total weekly aerobic exercise by 30 minutes vigorous or 60 minutes moderate	15.2 (13.0, 16.9)	-1.5 (-2.1, -0.9)	0.91 (0.88, 0.95)
Increase total weekly aerobic exercise by 45 minutes vigorous or 90 minutes moderate	14.6 (12.3, 16.4)	-2.1 (-2.9, -1.3)	0.87 (0.83, 0.92)

© 2026 Jayasekera J et al. *JAMA Network Open.*

Exercise strategy	8-year Risk, % (95% CI)	Risk difference, percentage points (95% CI)	Risk ratio (95% CI)
Increase total weekly aerobic exercise by 60 minutes vigorous or 120 minutes moderate	14.0 (11.8, 16.0)	-2.7 (-3.6, -1.6)	0.84 (0.79, 0.90)

[a]In each sequential target trial extension, an additional modification is added on top of all prior modifications. That is, under "Extension – Additionally allow eligible women to have a prior history of cancer," the target trial protocol is identical to that described for the first target trial, except that (i) eligible women can be diagnosed with stage 1 cancer and (ii) eligible women can have a prior history of cancer. Results for the extensions that additionally considered all-cause and breast cancer-specific mortality risks estimated over 10-years are shown in the main text in Table 3. [b]Under these strategies, eligible individuals in the target trial were required to achieve exercise levels similar to those of the health education or recreational aerobic exercise interventions of the CHALLENGE trial. Abbreviations: CI = Confidence Interval.

eTable 7. Sensitivity analysis changing the outcome to questionnaire non-responses (negative outcome control) - Estimated 10-year risks of questionnaire non-response under different tailored exercise strategies in the second target trial emulation using observational data from the Pathways Study (2005 – 2021)[a]

Exercise strategy	Questionnaire non-response		
	**10-year risk, % (95% CI)**	**Risk difference, percentage points (95% CI)**	**Risk ratio (95% CI)**
No intervention	68.3 (65.1, 71.0)	Reference	Reference
Increase total weekly aerobic exercise by 15 minutes vigorous or 30 minutes moderate	68.4 (65.1, 71.2)	0.0 (-0.4, 0.5)	1.00 (0.99, 1.01)
Increase total weekly aerobic exercise by 30 minutes vigorous or 60 minutes moderate	68.3 (65.0, 71.2)	-0.0 (-0.7, 0.7)	1.00 (0.99, 1.01)
Increase total weekly aerobic exercise by 45 minutes vigorous or 90 minutes moderate	68.1 (65.0, 71.3)	-0.2 (-1.1, 0.9)	1.00 (0.98, 1.01)
Increase total weekly aerobic exercise by 60 minutes vigorous or 120 minutes moderate	67.9 (64.8, 71.2)	-0.4 (-1.5, 1.0)	0.99 (0.98, 1.01)

[a]Strategies are tailored based on evolving individual characteristics. That is, individuals are no longer required to increase their aerobic exercise levels if and when they develop myocardial infarction, stroke, congestive heart failure, distant recurrence, or swelling that interferes with exercise.
Abbreviations: CI = Confidence Interval.

© 2026 Jayasekera J et al. *JAMA Network Open.*

eTable 8. Other sensitivity analyses - Estimated 10-year risks of all-cause and breast cancer-specific mortality under different tailored exercise strategies in the second target trial emulation using observational data from the Pathways Study (2005 – 2021)

Exercise strategy	All-cause mortality			Breast cancer-specific mortality		
	**10-year risk, % (95% CI)**	**Risk difference, percentage points (95% CI)**	**Risk ratio (95% CI)**	**10-year risk, % (95% CI)**	**Risk difference, percentage points (95% CI)**	**Risk ratio (95% CI)**
**Require eligible individuals to have a baseline weighted Elixhauser Comorbidity Index < 2**						
No intervention	18.2 (15.6, 20.7)	Reference	Reference	7.2 (5.5, 9.1)	Reference	Reference
Increase total weekly aerobic exercise by 15 minutes vigorous or 30 minutes moderate	17.4 (14.8, 19.8)	-0.8 (-1.2, -0.2)	0.95 (0.93, 0.99)	6.6 (5.0, 8.3)	-0.7 (-1.1, -0.3)	0.91 (0.85, 0.96)
Increase total weekly aerobic exercise by 30 minutes vigorous or 60 minutes moderate	16.7 (14.3, 19.2)	-1.5 (-2.2, -0.6)	0.92 (0.88, 0.97)	6.1 (4.5, 7.6)	-1.2 (-2.0, -0.5)	0.84 (0.73, 0.92)
Increase total weekly aerobic exercise by 45 minutes vigorous or 90 minutes moderate	16.0 (13.7, 18.7)	-2.2 (-3.1, -0.9)	0.88 (0.83, 0.95)	5.6 (4.1, 7.2)	-1.6 (-2.7, -0.7)	0.77 (0.64, 0.89)
Increase total weekly aerobic exercise by 60 minutes vigorous or 120 minutes moderate	15.5 (13.1, 18.3)	-2.7 (-4.0, -1.2)	0.85 (0.78, 0.93)	5.3 (3.6, 6.8)	-2.0 (-3.3, -0.9)	0.73 (0.57, 0.86)

© 2026 Jayasekera J et al. *JAMA Network Open*.

eTable 8. Other sensitivity analyses - Estimated 10-year risks of all-cause and breast cancer-specific mortality under different tailored exercise strategies in the second target trial emulation using observational data from the Pathways Study (2005 – 2021)

	All-cause mortality			Breast cancer-specific mortality		
**Exercise strategy**	**10-year risk, % (95% CI)**	**Risk difference, percentage points (95% CI)**	**Risk ratio (95% CI)**	**10-year risk, % (95% CI)**	**Risk difference, percentage points (95% CI)**	**Risk ratio (95% CI)**
**Require individuals to increase exercise until they develop a broader set of serious conditions**						
No intervention	21.2 (18.7, 23.1)	Reference	Reference	9.8 (7.9, 11.4)	Reference	Reference
Increase total weekly aerobic exercise by 15 minutes vigorous or 30 minutes moderate	20.3 (17.9, 22.1)	-0.9 (-1.4, -0.5)	0.96 (0.94, 0.97)	9.0 (7.4, 10.7)	-0.8 (-1.0, -0.3)	0.92 (0.90, 0.96)
Increase total weekly aerobic exercise by 30 minutes vigorous or 60 minutes moderate	19.5 (17.2, 21.4)	-1.7 (-2.3, -1.0)	0.92 (0.89, 0.95)	8.5 (6.9, 10.1)	-1.4 (-1.8, -0.6)	0.86 (0.82, 0.93)
Increase total weekly aerobic exercise by 45 minutes vigorous or 90 minutes moderate	19.0 (16.5, 20.8)	-2.3 (-3.3, -1.5)	0.89 (0.85, 0.93)	8.1 (6.5, 9.7)	-1.7 (-2.4, -0.8)	0.82 (0.76, 0.91)
Increase total weekly aerobic exercise by 60 minutes vigorous or 120 minutes moderate	20.3 (17.9, 22.1)	-0.9 (-1.4, -0.5)	0.96 (0.94, 0.97)	7.7 (6.1, 9.4)	-2.1 (-3.0, -1.0)	0.79 (0.71, 0.90)

© 2026 Jayasekera J et al. *JAMA Network Open.*

eTable 8. Other sensitivity analyses - Estimated 10-year risks of all-cause and breast cancer-specific mortality under different tailored exercise strategies in the second target trial emulation using observational data from the Pathways Study (2005 – 2021)

	**All-cause mortality**			**Breast cancer-specific mortality**		
**Exercise strategy**	**10-year risk, % (95% CI)**	**Risk difference, percentage points (95% CI)**	**Risk ratio (95% CI)**	**10-year risk, % (95% CI)**	**Risk difference, percentage points (95% CI)**	**Risk ratio (95% CI)**
**Require individuals to increase exercise until 2 years before they develop a serious condition**						
No intervention	18.6 (16.4, 21.0)	Reference	Reference	9.1 (7.4, 11.2)	Reference	Reference
Increase total weekly aerobic exercise by 15 minutes vigorous or 30 minutes moderate	18.5 (16.0, 20.5)	-0.2 (-0.9, -0.1)	0.99 (0.95, 0.99)	8.6 (6.9, 10.5)	-0.5 (-0.9, -0.2)	0.95 (0.91, 0.98)
Increase total weekly aerobic exercise by 30 minutes vigorous or 60 minutes moderate	17.9 (15.6, 20.1)	-0.7 (-1.6, -0.3)	0.96 (0.92, 0.98)	8.2 (6.7, 10.0)	-0.8 (-1.6, -0.4)	0.91 (0.84, 0.96)
Increase total weekly aerobic exercise by 45 minutes vigorous or 90 minutes moderate	17.6 (15.2, 19.8)	-1.1 (-2.2, -0.4)	0.94 (0.89, 0.98)	7.9 (6.3, 9.6)	-1.2 (-2.2, -0.5)	0.87 (0.78, 0.94)
Increase total weekly aerobic exercise by 60 minutes vigorous or 120 minutes moderate	17.3 (14.8, 19.7)	-1.3 (-2.7, -0.5)	0.93 (0.86, 0.97)	7.6 (6.1, 9.4)	-1.5 (-2.6, -0.6)	0.84 (0.73, 0.93)

© 2026 Jayasekera J et al. *JAMA Network Open*.

eTable 8. Other sensitivity analyses - Estimated 10-year risks of all-cause and breast cancer-specific mortality under different tailored exercise strategies in the second target trial emulation using observational data from the Pathways Study (2005 – 2021)

	All-cause mortality			Breast cancer-specific mortality		
**Exercise strategy**	**10-year risk, % (95% CI)**	**Risk difference, percentage points (95% CI)**	**Risk ratio (95% CI)**	**10-year risk, % (95% CI)**	**Risk difference, percentage points (95% CI)**	**Risk ratio (95% CI)**
**Additionally adjust for baseline income and HER2 status**						
No intervention	20.0 (17.5, 22.5)	Reference	Reference	8.7 (7.1, 10.4)	Reference	Reference
Increase total weekly aerobic exercise by 15 minutes vigorous or 30 minutes moderate	19.3 (16.7, 21.6)	-0.6 (-1.4, -0.4)	0.97 (0.93, 0.98)	7.9 (6.5, 9.7)	-0.8 (-1.2, -0.3)	0.91 (0.87, 0.96)
Increase total weekly aerobic exercise by 30 minutes vigorous or 60 minutes moderate	18.7 (16.0, 20.8)	-1.2 (-2.4, -0.7)	0.94 (0.89, 0.96)	7.4 (5.9, 9.2)	-1.3 (-1.9, -0.6)	0.85 (0.77, 0.93)
Increase total weekly aerobic exercise by 45 minutes vigorous or 90 minutes moderate	18.1 (15.4, 20.5)	-1.9 (-3.2, -1.0)	0.91 (0.84, 0.95)	7.0 (5.4, 8.9)	-1.7 (-2.5, -0.7)	0.81 (0.70, 0.92)
Increase total weekly aerobic exercise by 60 minutes vigorous or 120 minutes moderate	17.6 (14.9, 20.1)	-2.3 (-3.8, -1.1)	0.88 (0.81, 0.94)	6.7 (5.0, 8.7)	-2.0 (-3.0, -0.7)	0.77 (0.63, 0.91)

© 2026 Jayasekera J et al. *JAMA Network Open.*

eTable 8. Other sensitivity analyses - Estimated 10-year risks of all-cause and breast cancer-specific mortality under different tailored exercise strategies in the second target trial emulation using observational data from the Pathways Study (2005 – 2021)

	All-cause mortality			Breast cancer-specific mortality		
**Exercise strategy**	**10-year risk, % (95% CI)**	**Risk difference, percentage points (95% CI)**	**Risk ratio (95% CI)**	**10-year risk, % (95% CI)**	**Risk difference, percentage points (95% CI)**	**Risk ratio (95% CI)**
**Change the order in which time-varying covariates are modeled**						
No intervention	21.4 (18.8, 23.1)	Reference	Reference	9.9 (8.0, 11.6)	Reference	Reference
Increase total weekly aerobic exercise by 15 minutes vigorous or 30 minutes moderate	20.3 (17.8, 22.1)	-1.1 (-1.5, -0.6)	0.95 (0.93, 0.97)	9.1 (7.5, 10.7)	-0.9 (-1.2, -0.4)	0.91 (0.88, 0.96)
Increase total weekly aerobic exercise by 30 minutes vigorous or 60 minutes moderate	19.4 (17.0, 21.2)	-2.0 (-2.7, -1.1)	0.91 (0.88, 0.94)	8.5 (6.8, 10.0)	-1.5 (-2.1, -0.7)	0.85 (0.79, 0.92)
Increase total weekly aerobic exercise by 45 minutes vigorous or 90 minutes moderate	18.7 (16.1, 20.5)	-2.7 (-3.7, -1.6)	0.88 (0.83, 0.92)	8.0 (6.4, 9.5)	-2.0 (-2.9, -1.0)	0.80 (0.72, 0.89)
Increase total weekly aerobic exercise by 60 minutes vigorous or 120 minutes moderate	18.2 (15.4, 20.1)	-3.2 (-4.6, -1.9)	0.85 (0.79, 0.90)	7.5 (5.9, 9.3)	-2.4 (-3.5, -1.1)	0.76 (0.66, 0.88)

Abbreviations: CI = Confidence Interval.

© 2026 Jayasekera J et al. *JAMA Network Open*.

eTable 9. Estimates among women with any baseline level of exercise **-** Estimated 10-year risks of all-cause mortality and breast cancer-specific mortality under different tailored strategies in the second target trial emulation using observational data from the Pathways Study (2005 – 2021)[a]

	All-cause mortality			Breast cancer-specific mortality		
**Exercise strategy**	**10-year risk, % (95% CI)**	**Risk difference, percentage points (95% CI)**	**Risk ratio (95% CI)**	**10-year risk, % (95% CI)**	**Risk difference, percentage points (95% CI)**	**Risk ratio (95% CI)**
No intervention	17.7 (16.2, 19.1)	Reference	Reference	7.9 (6.6, 8.6)	Reference	Reference
Increase total weekly aerobic exercise by 15 minutes vigorous or 30 minutes moderate	17.3 (15.7, 18.5)	-0.4 (-0.9, -0.3)	0.98 (0.95, 0.99)	7.5 (6.4, 8.2)	-0.4 (-0.5, -0.2)	0.95 (0.93, 0.98)
Increase total weekly aerobic exercise by 30 minutes vigorous or 60 minutes moderate	16.8 (15.2, 18.0)	-0.9 (-1.5, -0.6)	0.95 (0.91, 0.97)	7.2 (6.0, 8.0)	-0.7 (-1.0, -0.3)	0.91 (0.87, 0.96)
Increase total weekly aerobic exercise by 45 minutes vigorous or 90 minutes moderate	16.5 (14.8, 17.7)	-1.2 (-2.1, -0.8)	0.93 (0.88, 0.96)	7.0 (5.7, 7.8)	-0.9 (-1.3, -0.4)	0.88 (0.82, 0.95)
Increase total weekly aerobic exercise by 60 minutes vigorous or 120 minutes moderate	16.2 (14.4, 17.4)	-1.5 (-2.5, -0.9)	0.91 (0.86, 0.95)	6.8 (5.5, 7.7)	-1.1 (-1.7, -0.4)	0.86 (0.78, 0.94)

[a]Strategies are tailored based on evolving individual characteristics. That is, individuals are no longer required to increase their aerobic exercise levels if and when they develop myocardial infarction, stroke, congestive heart failure, distant recurrence, or swelling that interferes with exercise.
Abbreviations: CI = Confidence Interval.

© 2026 Jayasekera J et al. *JAMA Network Open.*

eTable 10. Strategies requiring women to be insufficiently active, active, or highly active, as compared with minimally active - Estimated 10-year risks of all-cause and breast cancer-specific mortality under different exercise strategies, Pathways Study (2005 – 2021)[a]

	All-cause mortality			Breast cancer-specific mortality		
**Exercise strategy[b]**	**10-year risk, % (95% CI)**	**Risk difference, percentage points (95% CI)**	**Risk ratio (95% CI)**	**10-year risk, % (95% CI)**	**Risk difference, percentage points (95% CI)**	**Risk ratio (95% CI)**
Minimally active (< 100 MET-minutes/week)	36.0 (27.3, 44.7)	Reference	Reference	19.7 (13.3, 25.5)	Reference	Reference
Insufficiently active (100 – < 500 MET-minutes / week)	32.1 (26.3, 38.3)	-3.9 (-7.2, -0.7)	0.89 (0.83, 0.98)	16.5 (12.1, 20.6)	-3.2 (-5.8, -0.7)	0.84 (0.76, 0.95)
Active (500 – < 1,000 MET-minutes / week)	26.9 (23.2, 30.3)	-9.1 (-15.8, -1.8)	0.75 (0.63, 0.94)	12.6 (10.0, 15.1)	-7.1 (-12.3, -1.9)	0.64 (0.50, 0.86)
Highly active (≥ 1,000 MET-minutes / week)	19.8 (17.2, 21.7)	-16.2 (-24.6, -7.4)	0.55 (0.43, 0.74)	8.5 (6.8, 10.1)	-11.3 (-17.7, -4.9)	0.43 (0.31, 0.64)

[a]Strategies are not tailored based on any evolving individual characteristics. [b]Minimally active was defined as engaging in < 15 minutes of vigorous aerobic exercise / week or < 30 minutes of moderate aerobic exercise / week or an equivalent combination of vigorous and moderate aerobic exercise (< 100 MET-minutes / week). Insufficiently active was defined as engaging in 15 – < 75 minutes of vigorous aerobic exercise / week or 30 – < 150 minutes of moderate aerobic exercise / week or an equivalent combination of vigorous and moderate aerobic exercise (100 – < 500 MET-minutes / week). Active was defined as engaging in 75 – 150 minutes of vigorous aerobic exercise / week or 150 – 300 minutes of moderate aerobic exercise / week or an equivalent combination of vigorous and moderate aerobic exercise (500 – < 1,000 MET-minutes / week). Highly active was defined as engaging in ≥ 150 minutes of vigorous aerobic exercise / week, ≥ 300 minutes of moderate aerobic exercise / week, or an equivalent combination of vigorous and moderate aerobic exercise (≥ 1,000 MET-minutes / week). Abbreviations: CI = Confidence Interval; MET = Metabolic Equivalent of Task.

© 2026 Jayasekera J et al. *JAMA Network Open*.

eFigure 1. Flowchart of eligible individuals for the first target trial emulation using observational data from the Pathways Study (2005 – 2013[a]

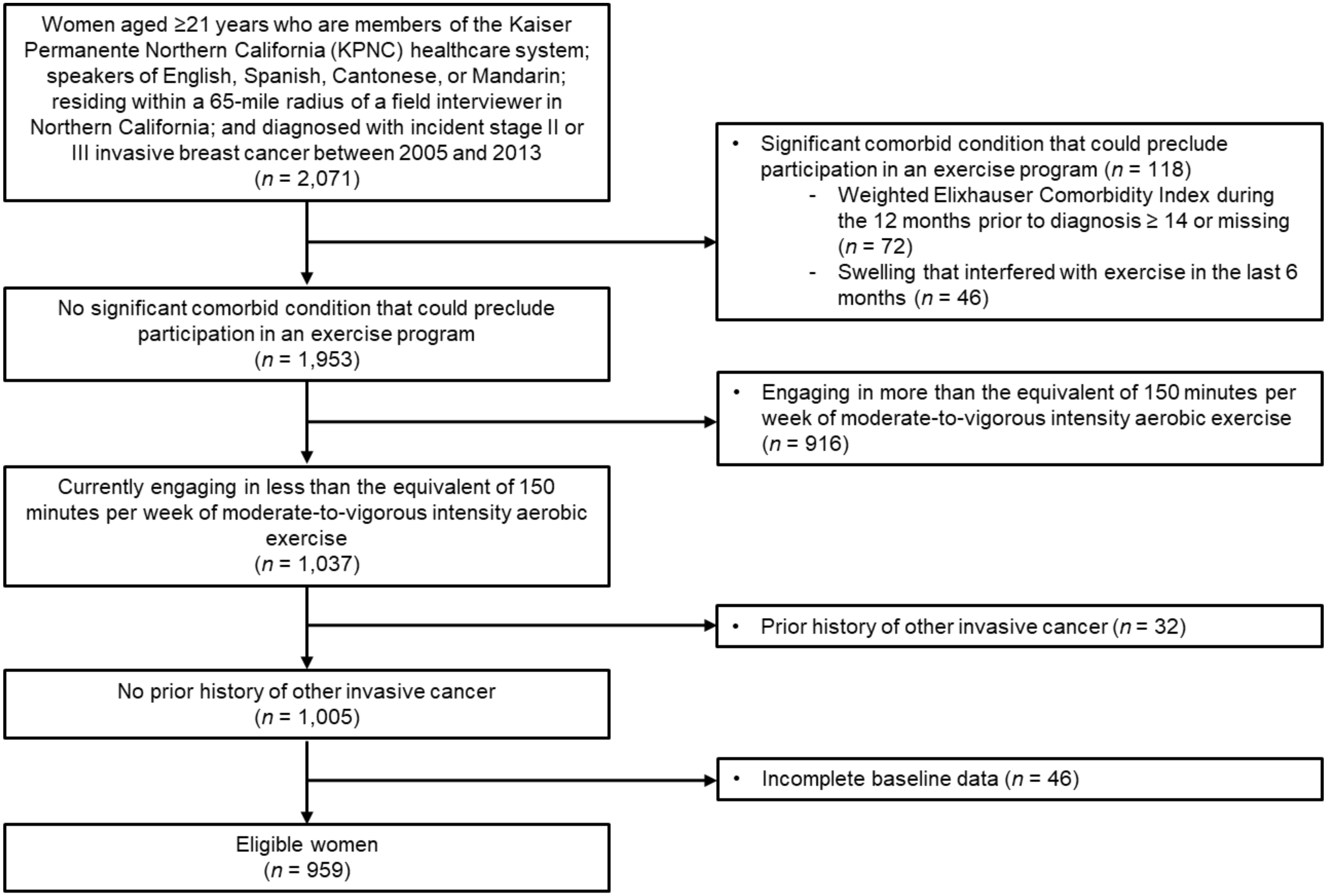

[a]Each eligible woman was assigned to all exercise strategies.

© 2026 Jayasekera J et al. *JAMA Network Open.*

eFigure 2. Flowchart of eligible individuals for the second target trial emulation using observational data from the Pathways Study (2005 – 2013)[a]

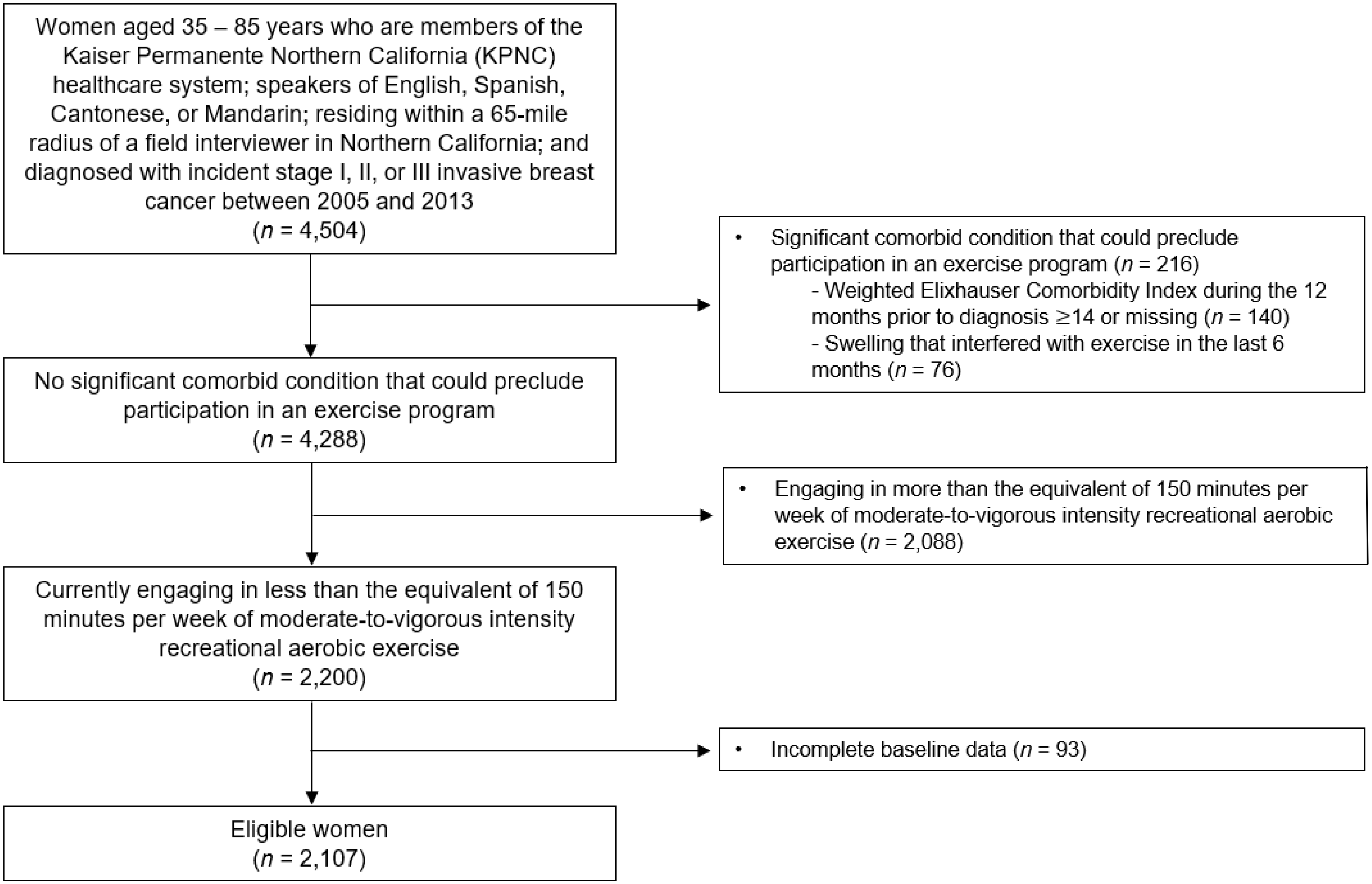

[a]Each eligible woman was assigned to all exercise strategies.

© 2026 Jayasekera J et al. *JAMA Network Open.*

eFigure 3. 10-year risk differences for all-cause and breast cancer-specific mortality comparing increases in aerobic exercise vs. no intervention (i.e., usual aerobic exercise) in the second target trial emulation using observational data from the Pathways Study (2005 - 2021)

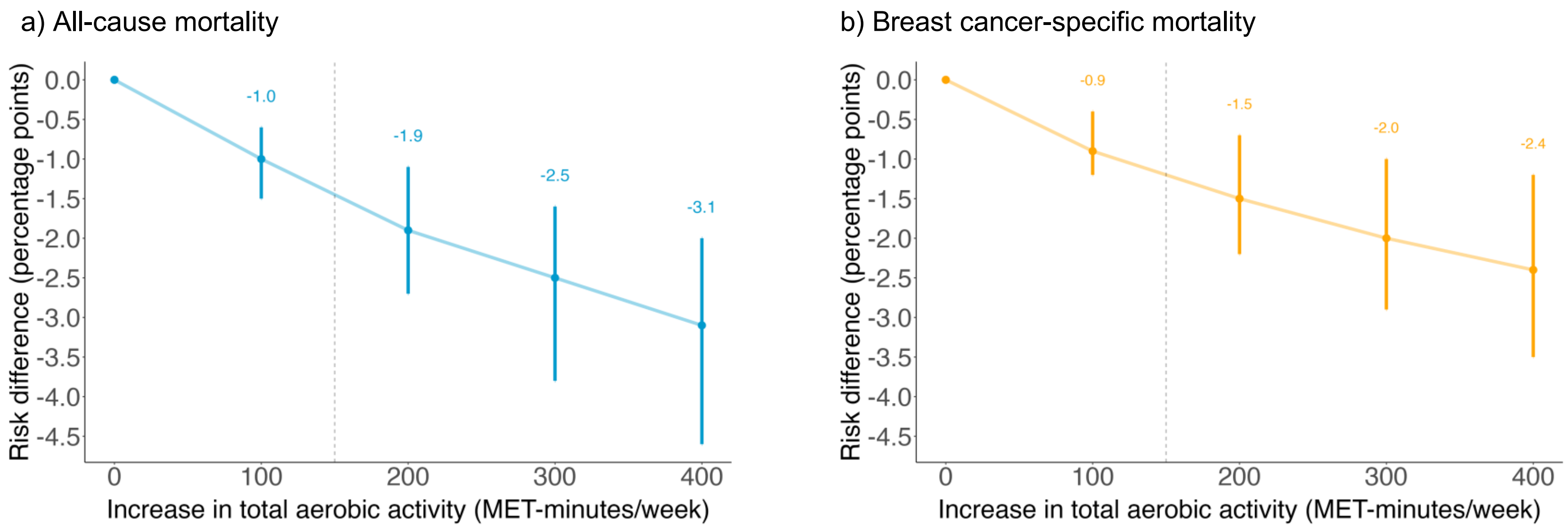

© 2026 Jayasekera J et al. *JAMA Network Open.*

eFigure 4. Comparison of inverse probability weighted vs. parametric g-formula estimates of means of the time-varying covariates under no intervention (i.e., usual exercise levels) in the second target trial emulation. Inverse probability weighted (solid red line) and parametric g-formula (dotted blue line) estimates are both adjusted for censoring due to loss to follow-up. The X-axis represents period of follow-up in 2-year units. The Y-axis represents the mean value or cumulative incidence of the variable.

a) Myocardial infarction, stroke, congestive heart failure, distant recurrence, or swelling that interferes with exercise (%)

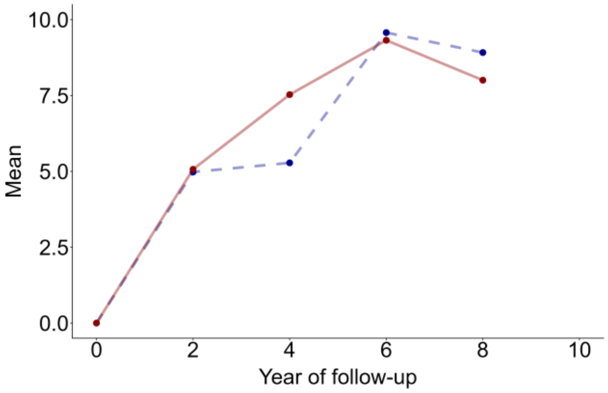

b) Body mass index (log(kg/m^2))

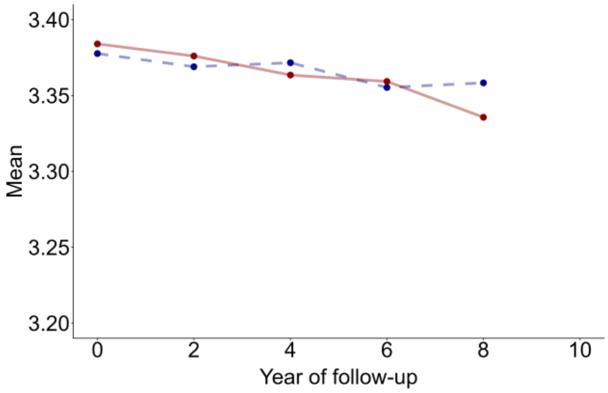

c) Muscle strengthening exercise (MET-minutes/week)

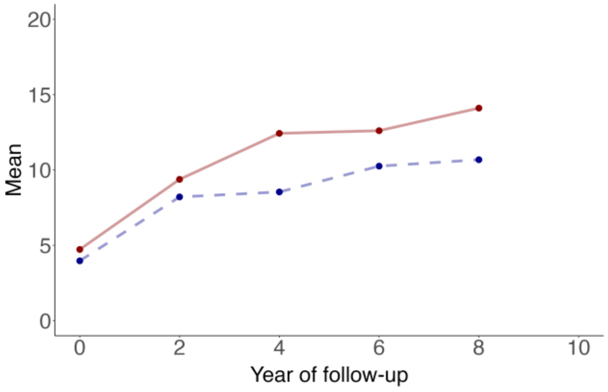

d) Total aerobic exercise (MET-minutes/week)

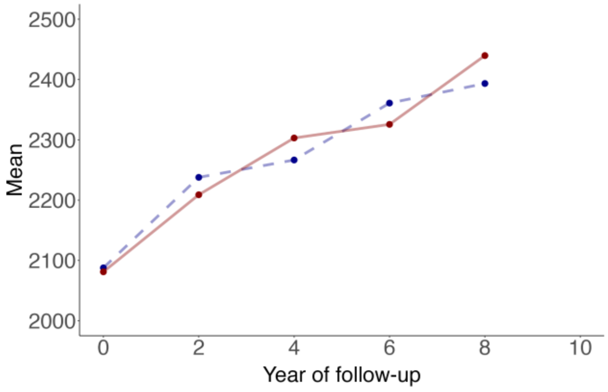

eMethods 2. TARGET checklist.

The TARGET (TrAnsparent ReportinG of observational studies Emulating a Target trial) Checklist[8]

Item no.		Checklist item		Location reported	
**Abstract**					
**1**	a	Identify that the study attempts to emulate a target trial using observational data. State the study objectives and briefly summarize the specified target trial.		Abstract (Design)	
	b	Report the data sources used for emulation.		Abstract (Design)	
	c	Summarize key assumptions, statistical methods, findings and conclusions.		Abstract (Design, Results, Conclusions and Relevance)	
**Introduction**					
**2**	Background	Describe the scientific background of the study and the gap in knowledge.		Introduction (first paragraph)	
**3**	Causal question	Summarize the causal question.		Introduction (second paragraph)	
**4**	Rationale	Describe the rationale for emulating a target trial with the available data. Cite randomized trials informing the design of the target trial if applicable.		Introduction (first and second paragraphs)	
**Methods**					
**5**	Data sources	Cite the data sources contributing to the analyses and for each one describe the following: original purpose, type, the geographic locations, setting and time-period. If relevant, describe how the data were linked or pooled.		Methods (Target Trial 1 – Target Trial Emulation section)	
**6**	**Target trial specification**<br>Specify the components of the target trial protocol that would answer the causal question.	**7**	**Target trial emulation**<br>Describe how the components of the target trial protocol were emulated with the observational data, including how all variables were measured or ascertained.	**Location item 6 (specification) reported**	**Location item 7 (emulation) reported**
	**Eligibility criteria**		**Eligibility criteria**		

© 2026 Jayasekera J et al. *JAMA Network Open.*

Item no.	Checklist item			Location reported	
a	Describe the eligibility criteria.	a	Describe how the eligibility criteria were operationalized with the data.	eTable 1	eTable 1
**Treatment strategies**		**Treatment strategies**			
b	Describe the treatment strategies that would be compared.	b	Describe how the treatment strategies were operationalized with the data.	eTable 1	eTable 1, eTable 2
**Assignment procedures**		**Assignment procedures**			
c	Report that eligible individuals would be randomly assigned to treatment strategies and may be aware of their treatment allocation.	c	Describe how assignment to treatment strategies was operationalized with the data.	eTable 1	eTable 1
**Follow-up**		**Follow-up**			
d	Clarify that follow-up would start at time of assignment to the treatment strategies. Specify when follow-up would end.	d	Clarify that follow-up starts at the time individuals were assigned to the treatment strategies. Describe how the end of follow-up was operationalized with the data.	eTable 1	eTable 1
**Outcomes**		**Outcomes**			
e	Describe the outcomes.	e	Describe how the outcomes were operationalized with the data.	eTable 1	eTable 1
**Causal contrasts**		**Causal contrasts**			
f	Describe the causal contrasts of interest, including effect measures.	f	Describe how the causal contrasts were operationalized with the data, including effect measures.	eTable 1	eTable 1
**Identifying assumptions**		**Identifying assumptions**			
g	Describe assumptions that would be made to identify each causal estimand. Describe the variables, if any, related to these assumptions.	g.i	For each causal estimand, describe assumptions made to identify it, including assumptions regarding baseline confounding due to lack of randomization.	eTable 1, Supplemental Methods I, eTable 3	eTable 1, Supplemental Methods I, eTable 3

© 2026 Jayasekera J et al. *JAMA Network Open*.

Item no.	Checklist item				Location reported	
			g.ii	Describe how the variables related to these assumptions were operationalized with the data		eTable 1, eTable 2
	**Data analysis plan**			**Data analysis plan**		
	h	For each causal estimand, describe the data analysis procedures and any associated statistical modelling assumptions, including approaches for handling missing data.	h.i	For each causal estimand, describe the data analysis procedures and any associated statistical modelling assumptions, including approaches for handling missing data.	eTable 1, Supplemental Methods I, eTable 3	eTable 1, Supplemental Methods I, eTable 3
			h.ii	For each causal estimand, describe any additional analyses conducted to assess the sensitivity of the results to the choice of operationalizations, assumptions and analysis.		eTable 1, eTable 4
**Results**						
**8**	Participant selection	Report numbers of individuals assessed for eligibility, eligible, and assigned to each treatment strategy. A flow diagram is strongly recommended.			eFigure 1, eFigure 2	
**9**	Baseline data	Describe the distribution of characteristics of individuals at baseline, by treatment strategy.			Table 1	
**10**	Follow-up	Summarize length of follow-up and describe reasons for end of follow-up for each treatment strategy and causal contrast.			Figure 1, Figure 2, eTable 5	
**11**	Missing data	Describe the frequency of missing data in all variables, by treatment strategy when applicable.			Supplemental Methods I	
**12**	Outcomes	Describe the frequency or distribution of each outcome, by treatment strategy.			Results	
**13**	Effect estimates	Report the effect estimates for each causal contrast with corresponding measures of precision, including both absolute and relative measures of effect, when applicable.			Results, Table 2, Table 3	
**14**	Additional analyses	Report results of all analyses to assess the sensitivity of the estimates to choices in operationalizations, assumptions and analysis.			eTable 7, eTable 8	
**Discussion**						
**15**	Interpretation	Provide an interpretation of the key findings.			Discussion	

© 2026 Jayasekera J et al. *JAMA Network Open.*

Item no.		Checklist item	Location reported
**16**	Limitations	Discuss the limitations of the study considering differences between the target trial and its emulation and the plausibility of assumptions, including assumptions regarding baseline confounding due to lack of randomization.	Discussion
**Other information**			
**17**	Ethics	Provide the institutional research board or ethics committee that approved the study and approval numbers, if relevant.	Methods (Target Trial 1 – Target Trial Emulation section)
**18**	Registration	State whether, when and where the study protocol was registered.	The study protocol was not registered
**19**	Sharing of study materials	Provide information on whether data, analytic code and/or other materials are accessible, and where and how they can be accessed.	Data Sharing Statement
**20**	Funding sources	Provide the sources of funding and detail the role of the funders in the design, conduct and reporting of the study.	Funding / Support and Role of the Funder / Sponsor
**21**	Conflicts of interest	State any conflicts of interest and financial disclosures for all authors.	Conflicts of Interest Disclosures

© 2026 Jayasekera J et al. *JAMA Network Open*.

eReferences

1. Robins JM, Hernán MA, Siebert U. Effects of multiple interventions. *Comparative Quantification of Health Risks: Global and Regional Burden of Disease Attributable to Selected Major Risk Factors (eds Ezzati, M, Lopez, A, Rodgers, A & Murray, C)*. World Health Organization; 2004.
2. Young JG, Herńan MA, Robins JM. Identification, estimation and approximation of risk under interventions that depend on the natural value of treatment using observational data. *Epidemiol Methods*. Dec 2014;3(1):1-19. doi:10.1515/em-2012-0001
3. Young JG, Vatsa R, Murray EJ, Hernán MA. Interval-cohort designs and bias in the estimation of per-protocol effects: a simulation study. *Trials*. Sep 5 2019;20(1):552. doi:10.1186/s13063-019-3577-z
4. Danaei G, Robins JM, Young JG, Hu FB, Manson JE, Hernán MA. Weight Loss and Coronary Heart Disease: Sensitivity Analysis for Unmeasured Confounding by Undiagnosed Disease. *Epidemiology*. Mar 2016;27(2):302-10. doi:10.1097/ede.0000000000000428
5. Lipsitch M, Tchetgen Tchetgen E, Cohen T. Negative controls: a tool for detecting confounding and bias in observational studies. *Epidemiology*. May 2010;21(3):383-8. doi:10.1097/EDE.0b013e3181d61eeb
6. Chiu YH, Wen L, McGrath S, Logan R, Dahabreh IJ, Hernán MA. Evaluating Model Specification When Using the Parametric G-Formula in the Presence of Censoring. *Am J Epidemiol*. Nov 3 2023;192(11):1887-1895. doi:10.1093/aje/kwad143
7. Courneya KS, Vardy JL, O'Callaghan CJ, et al. Structured Exercise after Adjuvant Chemotherapy for Colon Cancer. *N Engl J Med*. Jul 3 2025;393(1):13-25. doi:10.1056/NEJMoa2502760
8. Cashin AG, Hansford HJ, Hernán MA, et al. Transparent Reporting of Observational Studies Emulating a Target Trial—The TARGET Statement. *JAMA*. 2025;334(12):1084-1093. doi:10.1001/jama.2025.13350

© 2026 Jayasekera J et al. *JAMA Network Open*.
