# Supplementary material for: Tailored Exercise Strategies and Mortality Among Breast Cancer Survivors
Source: JAMA Netw Open. 2026 Apr 13;9(4):e265177. doi: 10.1001/jamanetworkopen.2026.5177 (PMC13077521; doi:10.1001/jamanetworkopen.2026.5177)
Supplement: Supplement 2. — Data Sharing Statement [file jamanetwopen-e265177-s002.pdf]

Data Sharing Statement

Jayasekera. Tailored Exercise Strategies and Mortality Among Breast Cancer Survivors. *JAMA Netw Open*. Published April 13, 2026. doi:10.1001/jamanetworkopen.2026.5177

Data
Data available: Yes
Data types: Deidentified participant data, Data dictionary
How to access data: A formal data request and data user agreement was used to obtain Kaiser Permanente Pathways Study data. Information for accessing this data can be found on the Pathways Study website. Further information is available upon request from the Kaiser Permanente Pathways Study authors.
When available: With publication

Supporting Documents
Document types: Statistical/analytic code
How to access documents: Statistical code used to generate the results presented in this paper is publicly available via GitHub: https://github.com/emma-mcgee/tailored-exercise-strategies-and-mortality.
When available: With publication

Additional Information
Who can access the data: Researchers whose proposed use of the data has been approved
Types of analyses: For any approved purpose
Mechanisms of data availability: After approval of a proposal
